# Pediatric Autoimmune Disorders Associated with Streptococcal Infections and Tourette's Syndrome in Preclinical Studies

**DOI:** 10.3389/fnins.2016.00310

**Published:** 2016-06-30

**Authors:** Chiara Spinello, Giovanni Laviola, Simone Macrì

**Affiliations:** Section of Behavioural Neuroscience, Department of Cell Biology and Neuroscience, Istituto Superiore di SanitàRoma, Italy

**Keywords:** PANDAS, Tourette's Syndrome, animal models, group A–beta hemolytic streptococcus, autoimmunity

## Abstract

Accumulating evidence suggests that Tourette's Syndrome (TS) – a multifactorial pediatric disorder characterized by the recurrent exhibition of motor tics and/or vocal utterances – can partly depend on immune dysregulation provoked by early repeated streptococcal infections. The natural and adaptive antibody-mediated reaction to streptococcus has been proposed to potentially turn into a pathological autoimmune response in vulnerable individuals. Specifically, in conditions of increased permeability of the blood brain barrier (BBB), streptococcus-induced antibodies have been proposed to: (i) reach neuronal targets located in brain areas responsible for motion control; and (ii) contribute to the exhibition of symptoms. This theoretical framework is supported by indirect evidence indicating that a subset of TS patients exhibit elevated streptococcal antibody titers upon tic relapses. A systematic evaluation of this hypothesis entails preclinical studies providing a proof of concept of the aforementioned pathological sequelae. These studies shall rest upon individuals characterized by a vulnerable immune system, repeatedly exposed to streptococcus, and carefully screened for phenotypes isomorphic to the pathological signs of TS observed in patients. Preclinical animal models may thus constitute an informative, useful tool upon which conducting targeted, hypothesis-driven experiments. In the present review we discuss the available evidence in preclinical models in support of the link between TS and pediatric autoimmune neuropsychiatric disorders associated with streptococcus infections (PANDAS), and the existing gaps that future research shall bridge. Specifically, we report recent preclinical evidence indicating that the immune responses to repeated streptococcal immunizations relate to the occurrence of behavioral and neurological phenotypes reminiscent of TS. By the same token, we discuss the limitations of these studies: limited evidence of behavioral phenotypes isomorphic to tics and scarce knowledge about the immunological phenomena favoring the transition from natural adaptive immunity to pathological outcomes.

## Introduction

Neuropsychiatric and neurological disorders are among the leading causes of disability worldwide (Silberberg et al., 2015). Several studies reported that people affected by neuropsychiatric illnesses show a set of psychosocial disturbances, ranging from difficulties in social interactions to emotional instability (Hartley et al., 2014; Cieza et al., 2015), ultimately resulting in difficulties in routine activities (Coenen et al., 2016). Since neuropsychiatric illnesses have a strong impact on the well-being of affected individuals, understanding the etiology of these diseases may beget remarkable heuristic advancements. Within this framework, epidemiological, clinical, and preclinical studies reveal that different determinants contribute to the pathogenesis of neuropsychiatric diseases. Among them, genetic factors (Hyman, 2008) and several environmental risk factors, such as prenatal and perinatal injuries or stressors (Bronson and Bale, 2016) and infectious phenomena (John et al., 2015), play a key role.

Autoimmunity, defined by Davison as “the failure of an organism to recognize its own part as self, resulting in a series of immunological responses to its own cells and tissues” (Davison, 2012) has emerged as a potential pathogenic factor in different types of neuropsychiatric illnesses, including autoimmune encephalitis (Höftberger, 2015), systemic lupus erythematosus (SLE; Podolska et al., 2015), or schizophrenia (Margari et al., 2013). Infectious phenomena constitute a vulnerability factor in the onset of autoimmune disorders. In particular, infections may trigger the onset of autoimmune diseases in the presence of vulnerability conditions. With respect to neuropsychiatric disorders, these vulnerability conditions are represented, for example, by an abnormal permeability of the blood brain barrier (BBB; Almutairi et al., 2016). Hornig (2013) proposed that microbes may contribute to the etiology of autoimmune neurological and neuropsychiatric disorders by triggering the production of autoantibodies that directly bind brain targets. In susceptible individuals, these phenomena can result in the appearance of behavioral and neurochemical abnormalities (Hornig, 2013).

Within this framework, streptococcal infections have been linked to a series of neuropsychiatric and movement disorders (Swedo et al., 1998). For example, different studies documented that the onset of Sydenham Chorea (SC), a variant of rheumatic fever, is linked to group A β-hemolitic streptococcus infections (Swedo et al., 1993; Cardoso et al., 1999). SC is characterized by choreiform movements that typically involve face and extremities and, in some cases, by behavioral difficulties and emotional liability (Swedo et al., 1989; Marques-Dias et al., 1997). Besides SC, several authors proposed that streptococcus infections may constitute an etiological factor also in a series of illnesses that typically arise during childhood. In particular, Swedo and colleagues proposed the acronym PANDAS (Pediatric Autoimmune Neuropsychiatric Disorder Associated with Streptococcal infections) to define a series of neurological and psychiatric disorders characterized by the presence of antibodies produced in response to group A β-hemolytic streptococcus infections (Swedo et al., 1998). The diagnostic criteria for PANDAS include: prepubertal onset; obsessive compulsive disorder (OCD) or chronic tic disorder; relapsing-remitting course of the disease; motor hyperactivity or reduced fine motor coordination; onset of the disease or symptoms exacerbation temporally related to streptococcal infection (Swedo et al., 1998).

In PANDAS and SC, antibodies produced in response to streptococcus have been proposed to be pathogenic in CNS in the context of an increased BBB permeability. Since the BBB is the primary protective barrier for neurons in central nervous system (CNS), BBB dysfunctions may contribute to the etiology of several neuropsychiatric disorders (Almutairi et al., 2016). In particular, in PANDAS and SC, after crossing the damaged BBB, cross-reactive antibodies may bind specific brain targets at the level of Basal Ganglia (BG), a brain structure involved in motor control (Martino et al., 2009; Murphy et al., 2010; Cutforth et al., 2016).

Streptococcal infections have also been suggested to relate to Tourette's Syndrome (TS), a multifactorial and complex disorder that may, in some cases, match the criteria for PANDAS (Hoekstra et al., 2013). TS is a childhood-onset disorder, in which chronic motor or phonic tics are the main symptoms. Tics are considered chronic if persist over a period longer than 12 months (Lombroso and Scahill, 2008). Tic, according to the DSM-5, is defined as “a sudden, rapid, recurrent, non-rhythmic motor movement or vocalization” (APA, 2013). TS is more frequent in males than females, with a ratio of 4:1. Typically, symptoms occur during prepubertal age, between 5 and 7 years, and have a waxing and waning course (Lombroso and Scahill, 2008). A gradual increase in tic frequency and severity is generally shown until 8–12 years, while a relevant reduction occurs in most patients at the end of adolescence (Leckman et al., 2010). Co-morbid conditions are typical in TS. In particular, obsessive-compulsive disorder (OCD) and attention-deficit/hyperactivity disorder (ADHD) are the most common comorbidities (Leckman et al., 2010). The pathogenesis of TS is multifactorial, and include genetic vulnerability (Deng et al., 2012), and several environmental risk factors such as prenatal and perinatal stressors or injuries and bacterial and viral infections (Leckman et al., 1987, 1990; Leckman and Peterson, 1993). With respect to precocious vulnerability, maternal factors (genetic or environmental) have been shown to increase individual vulnerability to TS. For example, Dalsgaard et al. (2015) recently reported that maternal autoimmune diseases significantly increase vulnerability to TS in the progeny (Dalsgaard et al., 2015).

While maternal autoimmunity can influence vulnerability to TS, it is yet to be determined whether these effects are genetic or environmental. With respect to genetic predispositions, several authors identified a series of genes for which a direct contribution to TS can be reasonably proposed. Thus, genetic linkage, cytogenetics and molecular genetic studies allowed identifying a set of genes potentially involved in TS (State, 2011). Among them, contactin-associated protein-like 2 (CNTNAP2), SLIT and NTRK-like 1 (SLITRK1) or membrane peptidase 2 like (IMMP2L) have been proposed as vulnerability genes. The proteic product of IMMP2L gene is a peptide with a catalytic function that, in the dysfunctional form, may cause the activation of the cell apoptotic mechanism through an aberrant mithocondrial functionality (Ma et al., 2011). Several authors reported in some members of a family with TS the presence of a translocation between chromosome 7 and 18 that causes the disruption of IMMP2L gene (Boghosian-Sell et al., 1996; Petek et al., 2001). However, the role of this gene in TS etiology remains unclear. CNTNAP2 is a transmembrane protein of the family of neurexin, abundantly expressed at the level of the axonal nodes of Ranvier, where it plays a crucial role in the cell-cell interaction. Poliak et al. (1999), hypothesized that this peptide may be involved in the positioning of K^+^ voltage-gated channel at the level of juxtaparanode region (Poliak et al., 1999). Verkerk et al. (2003) observed a chromosomal translocation between chromosome 2 and 7, in the region encoding CNTNAP2 protein, in a family of TS patients (Verkerk et al., 2003). The disruption of this region has been proposed to affect brain areas involved in motor control, thereby being responsible for the onset of tics (Verkerk et al., 2003). SLTRK1 is a member of a gene family that encodes a series of transmembrane proteins. The proteic product of SLTRK1 gene is a peptide that contains two leucine-rich repeat (LRR) motive and an intracellular C terminus having similarities with the tropomyosin-related kinase (Trk) neurotrophin's receptor (Aruga and Mikoshiba, 2003). SLTRK1 favors the formation of synapses, neuritic outgrowth and neuronal survival (Kajiwara et al., 2009). SLTRK1 transcription is regionally regulated in CNS; the pattern of expression is conserved among different mammalian species, such as mouse, rhesus monkey and human, and shows a preferential expression in brain areas involved in motor control, such as cortex, thalamus and basal ganglia (Stillman et al., 2009). In particular, SLRTK1 is expressed in the body compartment of cortex pyramidal projection neurons during adult life, and is preferentially associated, in the striatum, with neurons of the direct circuit expressing substance P and dopamine receptor D1, that project to substantia nigra (SN) and to globus pallidus (GP; Stillman et al., 2009). Some TS patients showed a missense mutation at the level of 3′ UTR of the SLTRK1 gene; this mutation leads to the production of a protein with an altered capacity of binding the microRNA 189 (Abelson et al., 2005). Moreover, an inversion in chromosome 13 in proximity of the region of the gene has been reported in patients with TS and ADHD (Proenca et al., 2011). Recently, Ercan-Sencicek et al. (2010) proposed that a mutation of the gene encoding for histidine decarboxylase (HDC) constitutes a rare genetic cause in TS (Ercan-Sencicek et al., 2010). In particular, the authors identified, through a study of a 2-generation pedigree in a family with a high incidence of TS, a rare segregating non-sense mutation in the *l-hystidine decarboxylase (hdc)* gene (Ercan-Sencicek et al., 2010). HDC is an enzyme necessary for the synthesis of histamine (HA) which, in turn, has been hypothesized to modulate DA level in CNS (Haas et al., 2008). Subsequently, a reduced concentration of HA in CNS (caused by the non-sense *hdc* gene mutation) may result in an altered dopaminergic regulation at the level of the basal ganglia circuitry, thereby resulting in TS symptomatology (Castellan Baldan et al., 2014). In the same study, Castellan Baldan and collaborators translated this evidence in an experimental model (*hdc* knock-out mice, see discussion for additional details). Moreover, an analysis of rare copy number variants in TS conducted on 460 patients, revealed the presence of a significant enrichment of genes involved in histaminergic pathways (Fernandez et al., 2012). In particular, the authors reported an enrichment in striatum and cortex of HA coupled G receptors H2 and H3. Those receptors are located both presinaptically and postsinaptically: presynaptic HA receptors are involved in the regulation not only of HA transmission, but also of dopamine (Fernandez et al., 2012). It is thus tenable to propose that dysfunctions in histaminergic pathway may contribute to the onset of TS through the modulation of dopaminergic transmission.

GAS infections, occurring after TS onset, have been proposed as a vulnerability factor potentially exacerbating symptoms (Martino et al., 2009; Landau et al., 2012). Additionally, in line with the possibility that altered immune capability constitutes a predisposing factor, clinical data support an increased vulnerability of the immune system in TS patients. For example, whilst Bos-Veneman et al. (2011) observed that TS children were characterized by decreased levels of IgG3 (Bos-Veneman et al., 2011), Kawikova et al. (2007) observed reduced concentrations of regulatory T cells in TS patients compared to controls (Kawikova et al., 2007). Moreover, during tic exacerbations, TS patients showed increased concentrations of cytokines, interleukin 12 (IL-12) and tumor necrosis factor alpha (TFN-α) in serum (Leckman et al., 2005; Martino et al., 2015). Several authors reported the presence of peripheral anti-streptococcal antibodies and anti-BG antibodies in patients affected by TS. For example, Cardona and Orefici observed that a large cohort of TS patients showed significantly higher levels of anti-streptococcal antibodies compared to control subjects; moreover, they reported that those patients had previously been exposed to streptococcal infections (Cardona and Orefici, 2001). Similarly, Rizzo and colleagues reported remarkably higher concentrations of anti-streptococcal antibody titers and a significantly higher presence of anti-BG antibodies in TS patients compared to control subjects (Rizzo et al., 2006). Martino and colleagues reported a similar increase in anti-BG antibodies in TS patients compared to controls (Martino et al., 2011).

Although these studies support the existence of a link between streptococcal infections and TS, several other studies failed to identify a direct link between immunization and TS symptoms (Singer et al., 2005a; Dale et al., 2006; Morris et al., 2009; Brilot et al., 2011). In particular, Singer et al. (2005a) performed ELISA and Western blot analyses against several epitopes present in the CNS (e.g., human postmortem caudate, putamen, prefrontal cortex) with sera obtained from PANDAS and TS patients, and controls. The authors did not detect differences in serum autoantibodies among groups (Singer et al., 2005a). Similarly, Morris et al. (2009), using a different experimental approach (immunofluorescence), failed to observe any difference among PANDAS and TS patients, and controls in terms of serum anti-striatal antibody reactivity (Morris et al., 2009). Finally, Brilot et al. (2011) reported the presence of serum autoantibodies capable of binding neuronal cell surface in SC patients, but not in PANDAS or TS patients (Brilot et al., 2011). These results demonstrate that the presence of autoimmune phenomena is neither a necessary nor a sufficient condition in the etiology of TS. However, the evidence discussed above indicates that a subset of TS cases may be dependent on autoimmune phenomena. Moreover, as already discussed, some cases of TS match criteria for PANDAS, suggesting that these two disorders may share — in specific circumstances — analogous etiopathological mechanisms.

Preclinical experimental models may constitute a valuable complement to clinical studies whereby they can aid the comprehension of the fundamental mechanisms favoring disease onset. Animal models may allow testing different hypotheses regarding the role exerted by variable factors in the onset and course of a given disease, and to design innovative therapeutic approaches (Rickard, 2004; van der Staay, 2006; van der Staay et al., 2009). Within this framework, several aspects of TS (symptomatology, genetic predisposition and environmental risk factors) have been translated into preclinical animal models (Hallett et al., 2000; Yaddanapudi et al., 2010; Brimberg et al., 2012; Macrì et al., 2015; see Macrì et al., 2013 for a detailed review).

Here, we will review preclinical data suggesting a link between autoimmunity and neurological diseases. In particular, we will discuss empirical evidence supporting the connection between TS and PANDAS, and the gaps of these studies that shall be filled in the future. Finally, in the light of the role of immunity in the onset of psychiatric disturbance, we discuss the possibility that peripheral autoantibodies may constitute an innovative biomarker of diagnostic use (Giana et al., 2015).

## Preclinical animal models and autoimmunity

Animal models constitute an important tool to aid the understanding of a given pathology and to potentially inform innovative therapeutic avenues. Thus, preclinical experimental models allow dissecting a given phenomenon into its fundamental determinants (e.g., genetic vs. environmental predisposing factors) and addressing the role that each of them plays, either in isolation or in combination with each other. The development of disease-related animal models rests upon several stages: the generation of a disease model based on a theoretical construct, the identification of abnormalities isomorphic to the symptoms observed in the patient population and the study of the efficacy of pharmacological treatments. The validity of each of these stages can be systematically scrutinized. Willner proposed three validity criteria: *construct, face*, and *predictive validity* (Willner, 1984).

*Construct validity* can be defined as the etiological similarity between the disease in human population and the experimental approach attempting to model such disease.

*Face validity* relates to the degree of similarity between the symptoms identified in the disorder examined and the phenotype (e.g., behavioral, physiological, immunological, neurobiological) in the experimental model (Willner and Mitchell, 2002). To fulfill this criterion, a valid animal model shall resemble the symptomatology observed in humans (van der Staay et al., 2009).

*Predictive validity* pertains to the therapeutic efficacy of available treatments. Specifically, to possess an elevated degree of predictive validity, a given experimental disease model shall be sensitive to the same available therapeutic approaches adopted in the patients (Willner, 1984).

Within this framework, the use of preclinical models has been extensively applied to the study of autoimmune neurological disorders (see Levite, 2014 and Hornig and Lipkin, 2013 for detailed reviews). Several preclinical animal models have been developed to address the link between circulating natural antibodies (directed against specific brain targets), and behavioral and neurochemical abnormalities. For example, mice immunized with GluR1 peptide fragments (a subunit of glutamate AMPA receptors) showed a significant elevation in circulating anti-GluR1 antibodies, marked hyperactivity, and increased self-grooming (Capone et al., 2008), the latter being associated with repetitive behavior (Kalueff et al., 2016). Also, mice immunized with dopamine transporter (DAT) fragments, displayed spontaneous hyperactivity, reduced cognitive flexibility and impulse control in operant behavioral paradigms. Moreover, the immunization protocol caused, as expected, an elevation in antibodies targeting dopamine transporter and a variation in brain striatal concentrations of dopamine and its metabolites (Adriani et al., 2012).

Glutamate is the main excitatory neurotransmitter in CNS (Platt, 2007) and is crucial for several neuronal functions. Abnormalities in glutamatergic neurotransmission have been shown to directly contribute to CNS disorders (Scoriels et al., 2015). The overactivation of glutamate receptors (excitotoxicity), induced by the excess of glutamate, may result in brain damage and neuronal death (Meldrum, 2000). Besides excitotoxicity, several types of anti-glutamate receptors antibodies are capable of inducing pathological effects in CNS (Levite, 2014). These autoantibodies emerged as one of the most widespread and dangerous pathogenic agents in CNS, causing impaired neuronal signaling and brain damages and contributing to the onset of a series of neuropsychiatric disorders (Levite, 2014). For example, patients affected by epilepsy and SLE, showed antibodies directed to different types of glutamate receptors, anti AMPA-GluR3B (Ganor et al., 2004, 2005a,b,c; Goldberg-Stern et al., 2014) and anti NMDA-NR2 (Borchers et al., 2005; Asano et al., 2013; Fanouriakis et al., 2013). From a translational perspective, antibodies against the same glutamate receptors have been shown to favor the onset of behavioral and neurochemical alterations also in preclinical models (see Levite, 2014 for a detailed review). These results have been observed in conditions of an increased permeability of the BBB (Kowal and Diamond, 2012). Several authors reported increased levels of anti-GluR3B antibodies in different mouse strains (specifically directed against peptide B of subunit R3 of glutamate AMPA receptors) after immunization with GluR3B peptide (Levite et al., 1999; Levite and Hermelin, 1999; Ganor et al., 2014). Specifically, Ganor et al. (2014) reported that DBA/2J mice (genetically epilepsy-prone mice) developed elevated titers of GluR3B antibodies after immunization with GluR3B peptide emulsified in Complete Freund's adjuvant (CFA). The presence of these antibodies aggravated seizures induced by the administration of a chemoconvulsant agent, and caused abnormal behaviors in mice. With respect to behavioral alterations, the authors observed, in mice positive to GluR3B antibodies, increased anxiety-like behaviors and motor impairments (problems in balance, motor coordination and muscle strength) compared to mice that did not show GluR3B antibodies in serum (Ganor et al., 2014).

Kowal and colleagues developed an immune-mediated mouse model of SLE (Kowal et al., 2004). These authors reported that the immunization of BALB/C mice with DNA peptide mimotope, arrayed as an octamer on a polylysine backbone (MAP peptide), induced the production of antibodies against subunit NR2 of NMDA glutamate receptor, associated with neuronal damages and cognitive impairments. In particular, following the administration of lipopolysaccharide (LPS, a procedure known to increase the permeability of the BBB) to immunized mice, NR2 antibodies bound neurons preferentially in hippocampus, inducing neuronal death and impaired memory (Kowal et al., 2004). NMDA-NR2 receptors are expressed throughout the brain, but at highest density within hippocampus, hypothalamus, and amygdala. When the BBB damage was induced in mice by epinephrine administration, Huerta et al. (2006) showed that anti-NR2 antibodies bound preferentially amygdala's neurons. Accordingly, immunized mice showed alteration in emotional behavior whereby they responded deficiently to fear-conditioning paradigms (Huerta et al., 2006). The latter has been shown to depend on an intact functionality of the amygdala (Sengupta et al., 2016).

Beside glutamate receptors, autoimmune phenomena in CNS involve other receptors, such as leucine-rich glioma inactivated 1 (LGI1), acquaporin-4 (AQP4), Gamma-Amino Butyric Acid (GABA_B_), or myelin oligodendrocyte protein (MOG; see Irani et al., 2014 for a detailed review). In preclinical studies, immunization with MOG has been shown, in susceptible animals, to trigger the onset of a series of inflammatory diseases and thereafter named experimental autoimmune encephalomyelitis (EAE). EAE, considered as a valid animal model of multiple sclerosis (MS), are a group of pathologies characterized by neurodegeneration, extensive inflammation and demyelination in CNS. These neurochemical alterations cause severe progressive motor impairments that ultimately result in flaccid paralysis of hind limbs (see Kipp et al., 2012 for a detailed review). Beside MOG (Amor et al., 1994, 1996), other myelin antigens are capable of triggering the onset of EAE in rodents, such as myelin basic protein (MBP, see Swanborg, 2001 and Amor et al., 1996), proteolipid protein (PLP, see Amor et al., 1993 and Amor et al., 1996) in presence of increased BBB permeability (Rabchevsky et al., 1999). Several preclinical studies showed that EAE are associated not only with severe motor deficits, but also with behavioral and cognitive impairments (Mandolesi et al., 2010; Acharjee et al., 2013; Olechowski et al., 2013). For example, Mandolesi et al. (2010) reported that EAE mice, compared to controls, showed hippocampal-dependent deficit in learning and memory (Mandolesi et al., 2010). Similarly, Acharjee and colleagues observed that EAE mice exhibited cognitive and behavioral impairments in a precocious phase of the disease (Acharjee et al., 2013). In particular, EAE mice exhibited reduction in the time spent in the target quadrant of Morris Water maze and impaired memory extinction in a fear-conditioning paradigm (Acharjee et al., 2013). Regarding behavioral impairments, authors observed in EAE mice increased anxiety-like behaviors. EAE mice showed, compared to controls, more time in the marginal zone of the apparatus during open field test and increased time in the closed arm of plus maze test (Acharjee et al., 2013). Finally, Olechowski et al. (2013) observed impairments in cognitive processes (assessed with a novel object recognition test) in a precocious phase of the disease (Olechowski et al., 2013).

The experimental evidence described above suggests that autoimmune phenomena against CNS targets may trigger, in vulnerability conditions, the development of remarkable phenotypic abnormalities. The appearance of different behavioral and neurochemical impairments depends on the brain target affected by the autoimmune phenomena and, in some instances, by the tools adopted to modulate BBB integrity. As reported above (see Introduction), analogous mechanisms have been proposed to contribute to the onset and exacerbation of streptococcal-related motor disturbances in clinical populations. Specifically, several authors (Martino et al., 2009; Murphy et al., 2010) proposed that antibodies produced in response to streptococcal infections (STREP) may, in presence of an increased vulnerability of the BBB, induce a pathological phenotype. In particular, these authors proposed that STREP-related antibodies may cross the damaged BBB and bind specific brain targets at the level of Basal Ganglia (BG), a brain structure involved in motor control. This cascade of events may ultimately provoke a symptomatology typical of streptococcal-related motor disturbances. In the next section we will describe some specific animal models developed with the aim of dissecting the mechanisms bridging the immunologic responses to streptococcal infections to the onset of neurological and behavioral dysfunctions.

### A potential link between streptococcal infections and TS

#### Passive transfer of sera from TS patients

Different approaches have been used to develop animal models addressing the role of streptococcal infections in immune-mediated neuropsychiatric disorders (Table 1). The first line of studies entailed direct intracerebral administration, in rats, of anti-neuronal antibodies sampled from TS patients (Hallett et al., 2000; Taylor et al., 2002; Loiselle et al., 2004; Singer et al., 2005b; Ben-Pazi et al., 2012; Yeh et al., 2012). Hallett et al. (2000) showed that brain intra-striatal microinfusions of TS sera induced behavioral stereotypies and episodic utterances (EU, repetitive, medium pitched sound of short duration) in male Fischer 344 rats. Stereotypies and EUs are considered analogous to involuntary movements observed in TS patients. In particular, the authors performed two separate studies, microinfusing either sera obtained from TS children or Gamma Immunoglobulin (IgG) isolated from these sera. In the first study, compared to facility-reared controls, rats microinfused with TS-sera showed exacerbated licking behavior, forepaw shaking and EU. Those abnormal behaviors were present during microinfusion, and on days 8–10 after the end of microinfusion, when behavior was assessed. EUs were particularly interesting, since rats usually do not emit audible vocalizations in non-threatening environment (Kaltwasser, 1990), as the one adopted in this study. The authors proposed that sudden and involuntary contraction of respiratory muscles (resulting from the effect of serum on rats' striatal functionality) may provoke EU. Furthermore, these sudden and audible vocalizations occurred in association with head or oral stereotypies, suggesting that contraction of respiratory muscles could be involved in their occurrence (Ebrahimi et al., 1992). In the second study, IgG was isolated from both control and TS sera and microinfused into rats' striatum. Compared to control individuals, rats microinfused with TS-IgG exhibited a much higher level of licking activity. Moreover, immunohistochemical analyses of brain sections showed that TS-IgG selectively bind striatal neurons in rats microinfused with TS-IgG. These results supported the hypothesis that IgG recognize specific neuronal antigens within the striatum and interfere with its normal functioning, inducing abnormalities in motor control (Hallett et al., 2000). Similarly, Taylor et al. (2002) observed an increase in oral stereotypies after infusions of TS sera into the ventrolateral striatum of male Sprague-Dawley rats. In this study, the authors performed 5 days of microinfusions and conducted systematic behavioral observations throughout the entire treatment period. Specifically, the authors assessed the behavior of three groups of rats, with different sera received during microinfusion: TS-sera containing high levels of autoantibodies, TS-sera containing low levels of autoantibodies and control sera. Rats microinfused with TS-sera characterized by highest antibody titers exhibited remarkably elevated oral stereotypies compared to the other groups (Taylor et al., 2002). Finally, Yeh et al. (2012) showed that TS sera were immunoreactive against a 120 kDa protein, identified as hyperpolarization-activated nucleotide channel 4 (HCN4) protein. Male Sprague-Dawley rats received microinfusion in striatum of purified anti-HCN4 antibodies and of TS-sera. Behavioral observation after infusion revealed that both TS sera and purified anti-HCN4 antibodies induced the increase of behavioral stereotypies in rats in a dose-dependent manner (Yeh et al., 2012). Additionally, several studies reported that passive transfer, in striatum of naïve animals, of anti-streptococcal sera or of purified IgG from animals exposed to GABHS, leads to the onset of behavioral and neurochemical abnormalities that resemble PANDAS symptomatology (Yaddanapudi et al., 2010; Lotan et al., 2014). These studies are detailed in the next section.

**Table 1:** **Summary of the main findings in TS/PANDAS animal models**.

**References**	**Passive transfer (PT) or direct immunization (DI)**	**Reference pathology**	**Neurochemical/Neuroanatomical abnormalities**	**Behavioral outcome**
Hallett et al., 2000	PT of sera or purified IgG from TS patients	TS	IgG deposits in striatum	(↑) Licking (↑) Forepaw shacking (↑) Episodic utterances
Taylor et al., 2002	PT of sera from TS patients	TS	–	(↑) Oral stereotypies
Yeh et al., 2012	PT of sera or purified anti-HCN4 from TS patients	TS	–	(↑) Stereotyped tic behavior
Hoffman et al., 2004	DI	TS/PANDAS	IgG deposits in deep cerebellar nuclei (DCN)	(↑) Rearing behavior
Yaddanapudi et al., 2010	DI	TS/PANDAS	IgG deposits in cerebellum and striatum	(↓) Social activities and social investigation (↓) Ability in olfactory discrimination (↓) Motor coordination (↓) Aggressive behavior (↑) Rearing behavior
	PT of sera from immunized mice	TS/PANDAS	IgG deposits in hippocampus and paraventricular area	(↑) Rearing behavior (↓) Social interaction
Macrì et al., 2015	DI	TS/PANDAS	Inflammation in rostral diencephalon	(↓) Pre-pulse inhibition (↑) Perseveration
Brimberg et al., 2012	DI	TS/PANDAS	IgG deposits in striatum, thalamus and frontal cortex	(↓) Motor capacity (↑) Compulsive behavior
Lotan et al., 2014	DI	TS/PANDAS	–	(↑) Rearing behavior (↓) Food manipulation (↓) Fine motor coordination
	PT of purified IgG from immunized rats	TS/PANDAS	Co-localized IgG deposits with D1/D2 receptor and SERT in striatum	(↓) Food manipulation (↓) Fine motor coordination

*Summary of the main articles in which an autoimmune hypothesis of motor disturbances has been addressed. We report the immunization method, the reference pathology, the neuroanatomical, neurochemical, and behavioral alterations identified*.

Although these results support the existence of a link between autoimmune phenomena and behavioral stereotypies, analogous subsequent studies failed to replicate these findings (Loiselle et al., 2004; Singer et al., 2005b; Ben-Pazi et al., 2012). In particular, Loiselle et al. (2004) performed microstriatal infusions of serum from TS and PANDAS patients in Fischer rats' striatum. In this study, rats received bilateral microinfusions of sera in ventral and ventrolateral striatum. As in the experimental protocol described in Hallett et al. (2000), sera were microinfused for 3 days, and animal behavior was assessed during microinfusions and for 10 days after the end of microinfusions. Unlike the two studies previously described (Hallett et al., 2000; Taylor et al., 2002), rats microinfused with TS sera or PANDAS sera did not show a significant increase in terms of motor or vocal stereotypies (Loiselle et al., 2004). Similarly, Singer et al. (2005b) observed that infusions of sera from patients with TS in ventrolateral striatum of Sprague-Dawley rats, did not significantly increase stereotypies (Singer et al., 2005b). A total of 16 rats received (for 4 days) sera containing elevated or low levels of antineural antibodies (ANAb), while eight control rats were infused with phosphate buffered saline (PBS). Behavioral observations were performed for 3 days before infusions, on days 2–4 during infusions, and for 3 days after the end of infusions. Stereotypies resulted significantly increased after serum infusion, but authors did not observe significant differences between control group and groups treated with low or elevated ANAb sera. Moreover, in contrast with Taylor et al. (2002), this study suggests that the level of antibodies in blood may have no influence on their pathogenicity; low or elevated titers of antineural antibodies in sera did not induce a differential behavioral response in terms of stereotypies (Singer et al., 2005b). Finally, Ben-Pazi et al. (2012), did not observe motor behavioral changes in rats after the injections of sera from Sydenhams's Chorea (SC) patients (Ben-Pazi et al., 2012). In particular, authors injected stereotaxically 6 μl of the IgG fraction of serum in rats' left striatum, and induced rotational behavior administering amphetamine and apomorphine (after 10 and 17 days from injections respectively). Authors observed that the injections of SC-IgG in rats brain striatum did not induce a significant increase in rotational behavior. Moreover, immunohistology staining, specific for dopaminergic or GABA-ergic markers, did not reveal cellular changes in rats injected with SDC-IgG compared to controls (Ben-Pazi et al., 2012). Although the reason for failure to detect stereotypies is unclear, Loiselle et al. (2004), proposed that methodological variations may constitute a possible explanation for the variable results obtained among different studies. These variations comprise different methods to quantify antineural antibodies in sera, strain of rodents, timing of microinfusion, timing of observation, and concentrations of microinfused sera (Loiselle et al., 2004).

#### Active immunization with group A beta–hemolytic streptococcus homogenate

Other experimental studies, using a different approach based on active immunization, reported that streptococcal infections may trigger, in the presence of a vulnerable BBB, basal ganglia dysfunctions (Swerdlow and Sutherland, 2005). These studies show that streptococcus exposure may favor the onset of behavioral disturbances and neurochemical alterations, thereby providing additional information regarding PANDAS etiology (Hoffman et al., 2004; Yaddanapudi et al., 2010; Brimberg et al., 2012; Macrì et al., 2015). These results may support the hypothesis that antibodies produced in response to streptococcus infections may bind, in a context of BBB permeability, brain targets at the level of basal ganglia, causing the onset of behavioral and motor disturbances and neurochemical alterations (Martino et al., 2009).

For example, SJL/J mice (a mouse strain prone to the induction of autoimmune encephalitis, see Korngold et al., 1986) repeatedly immunized with a group A beta—hemolytic streptococcus (GABHS) homogenate emulsified in Freund's adjuvant (FA), showed increased behavioral abnormalities compared to control subjects immunized with FA alone (Hoffman et al., 2004). Mice were screened in several behavioral tests to assess anxiety-like behavior, general behavioral responses, and exploratory behavior. Moreover, sera from all mice were tested for immunoreactivity to mouse brain, while the presence of IgG deposits has been assessed performing immunohistochemistry on cerebral tissues. The authors reported that a subset of sera collected after the second boost from GABHS mice were immunoreactive to several brain regions. In particular, GABHS sera labeled neurons in deep cerebellar nuclei (DCN), globus pallidus, and thalamus. GABHS immunized mice, characterized by serum immunoreactivity to DCN, showed also IgG deposits in the same brain area. Mice that showed serum immunoreactivity to DCN exhibited also increased rearing behavior (considered as repetitive behavior) compared to control mice and to GABHS subjects that did not show sera immunoreactivity to DCN. Moreover, the increase in rearing behavior correlated with DCN IgG deposits, and with serum IgG immunoreactivity to GABHS proteins. These results partially fulfill the criteria for PANDAS proposed by Swedo et al. (1998). In particular, the animal model described meets two criteria: the presence of chronic tic disorder and/or OCD; and the onset and exacerbation of symptoms associated with GABHS infections. Mice exposed to GABHS showed abnormal repetitive behavior, partially reproducing OCD symptomatology in humans. Moreover, the exhibition of repetitive behavior was temporally related with the exposure to GABHS (Hoffman et al., 2004).

In a subsequent study, Yaddanapudi et al. (2010) showed that humoral immunity is necessary and sufficient to induce PANDAS related symptoms. The authors passively immunized SJL mice by exposing them to serum obtained from donor mice immunized with GABHS homogenate, and observed an abnormal behavioral phenotype (Yaddanapudi et al., 2010). Direct exposure to GABHS homogenate resulted in diminished motor coordination, increased rearing behavior, reduced social activities and social investigation, inhibition of aggressive behavior and impaired ability in olfactory discrimination. Passive transfer of GABHS sera reproduced the increment in rearing behavior and the alteration in social interaction, while did not have effects on motor coordination. To demonstrate that the effects were due to the immune response to the streptococcus immunization, the authors also performed a passive transfer study in which sera of donor mice was depleted from Immunoglobulin G. IgG emerged as the active component of GABHS donor sera whereby its depletion abolished the behavioral abnormalities observed in mice injected with non-depleted IgG GABHS sera. Consistently with what emerged regarding behavioral observations, donor GABHS mice showed brain IgG deposits in cerebellum and striatum and mice injected with non-depleted IgG GABHS sera showed brain IgG deposits in hippocampus and paraventricular area. Conversely, IgG-depleted GABHS mice did not show brain deposits, confirming that IgG is the active component of GABHS sera. The different localization of brain IgG deposits in donor mice and in mice that received non-depleted sera, may depend on the different approaches used to increase the permeability of BBB (Freund's adjuvant and LPS respectively). These results, together with what observed in experimental studies involving animal models of SLE (see Kowal et al., 2004 and Huerta et al., 2006), suggest that BBB permeability is crucial in mediating the involvement of peripheral immunity in PANDAS and, in general, in neuropsychiatric and neurological disorders (Almutairi et al., 2016). Recently, Dileepan et al. (2016), proposed a mechanism that allows antibodies produced in response to streptococcal infections to cross the BBB and trigger autoimmune diseases of the CNS (Dileepan et al., 2016). In particular, they reported the presence of group A streptococcal specific Th17 lymphocytes in tonsils of humans previously exposed to natural GABHS infections (Dileepan et al., 2016). Repeated intranasal (i.n.) inoculations of GABHS in mice triggered the expansion of Th17 cells and the production of interleukin 17 (IL17), as shown in a previous study (Dileepan et al., 2011). IL17 causes the damaging of BBB barrier through the production of reactive oxygen species (ROS) in endothelial cells (Kebir et al., 2007; Huppert et al., 2010). Dileepan et al. (2016) repeatedly inoculated mice i.n. with GABHS to investigate if exposure to streptococcus induces Th17 GABHS-specific cells enter the mice brain. They reported that group A streptococcal infections trigger in mice a lymphocyte Th17 response together with the production of IL-17A in nasal-associated lymphoid tissue (NALT). NALT is a tissue located in proximity of cribriform plate and has an equivalent functionality of palatine tonsils in humans (Park et al., 2003). Moreover, they reported the presence of GABHS-specific Th17 cells associated with damaged BBB; the damaged BBB allowed the deposition of serum IgG. Finally they reported the presence of activated microglia (neuroinflammation) and impaired synaptic transmission. The authors suggested that the abnormal production of cytokine induced by infections may disrupt the BBB, permitting autoantibodies to access the brain and bind neural targets, ultimately causing the onset of pathological phenotypes (Dileepan et al., 2016).

Recently, we repeatedly exposed developing male SJL/J mice to a GABHS homogenate, showing that a single exposure to streptococcus is not sufficient to trigger behavioral abnormalities related to PANDAS (Macrì et al., 2015). In particular, we exposed mice to a primary immunization (GABHS homogenate emulsified in CFA), followed by three boosts (GABHS homogenate emulsified in incomplete Freund's adjuvant). We screened mice in two different behavioral test batteries performed between the primary immunization and the first boost, and after the second boost. Mice exposed to a single GABHS immunization did not show a differential phenotype compared to controls. Conversely, after the second boost, GABHS mice showed increased repetitive and perseverative behaviors and impaired sensorimotor gating. To evaluate sensorimotor gating, we measured their motor response in the Prepulse Inhibition of the startle reflex (PPI) task. PPI is an experimental measure in which the startle reflex (response to sudden and intense stimulus) is inhibited by a weak stimulus. This task is of common use in human laboratory and holds an elevated translational value (Swerdlow, 2013). In rodents, whole-body startle is measured by assessing the force resulting from the contraction of skeletal muscles (Swerdlow, 2013). PPI results impaired in a series of neuropsychiatric disorders, including schizophrenia (Swerdlow et al., 2006), Huntington disease (Swerdlow et al., 1995; Valls-Sole et al., 2004), OCD (Swerdlow et al., 1993; Hoenig et al., 2005; Ahmari et al., 2012), as well as TS (Castellanos et al., 1996; Swerdlow et al., 2001a,b; Zebardast et al., 2013). Preclinical evidence showed that in rodents experimental lesions of striatal circuits significantly reduced PPI (Baldan Ramsey et al., 2011), and that the administration of dopaminergic drugs modulated its expression (Mansbach et al., 1988; Russig et al., 2004). Therefore, impaired PPI observed in GABHS mice supports the hypothesis that repeated exposures to streptococcus may cause dysfunctions in cortico-striatal-thalamocortical (CSTC) circuits, involved in TS (Swerdlow, 2013). A dysfunctional regulation of the CSTC has been proposed to constitute a common factor among TS and comorbid problems, such OCD (Berardelli et al., 2003; Leckman et al., 2010). This hypothesis is supported by clinical evidence suggesting the involvement of the central dopaminergic system in TS: tics frequency is increased by dopamine (DA) D2 receptor agonists (Shprecher and Kurlan, 2009), and reduced by D2 antagonists (Scahill et al., 2006).

The increased perseverative behavior observed in GABHS mice constitutes an additional evidence supporting the hypothesis that repeated exposure to streptococcus may cause dysfunctions in brain areas considered involved in TS. In particular, we assessed perseverative behavior in T-maze test to measure spontaneous alternation, considered as a natural tendency to explore the environment (Deacon and Rawlins, 2006). Lalonde (2002) showed that the exhibition of spontaneous alternation depends on the integrity of several brain areas, including prefrontal cortex and dorsal striatum (Lalonde, 2002). Moreover, the administration of dopaminergic and serotoninergic drugs modulates spontaneous alternation behavior (Irwin et al., 1983; Jaffard et al., 1991).

The fact that behavioral abnormalities have been observed after repeated exposures to GABHS supports the hypothesis that a single immunization with streptococcus is not sufficient to trigger a pathological phenotype. The exhibition of symptoms may require a prolonged exposure, associated with the development of a high level of peripheral anti-GABHS antibodies. This hypothesis is supported by the fact that we found elevated concentrations of antibodies in GABHS-mice sera after repeated injections, but not after the primary immunization. Mice repeatedly exposed to streptococcus showed also neurochemical alterations (reduced serotonin and increased lactate) in prefrontal cortex, a brain structure involved in the control of the behavioral domains addressed in the study. Moreover, GABHS mice exhibited inflammatory processes (presence of infiltrates and active microglia) in the rostral diencephalon. Thus, our study supports the hypothesis that exposure to streptococcus is a vulnerability factor in the onset of behavioral and neurochemical phenotypes homologous to symptoms observed in PANDAS.

Brimberg et al. (2012) reported that male Lewis rats exposed to GABHS antigens, showed behavioral, immunological, and neural characteristics resembling symptoms observed in PANDAS patients (Brimberg et al., 2012). Rats exposed to GABHS exhibit impaired motor capacity and compulsive behavior. The administration of haloperidol and paroxetine, both used to treat motor symptoms and compulsion in PANDAS, alleviated symptoms observed in GABHS mice. Importantly, this study was the first reporting the presence of peripheral autoantibodies against D1 and D2 receptors following active immunization with GABHS homogenate. Moreover, GABHS-exposed rats showed IgG deposits in striatum, thalamus and frontal cortex. This study supports the link between GABHS exposure and the development of anti-brain antibodies (in rats sera), specifically directed against dopamine receptors. This evidence supports the idea that dopaminergic system has an important role in the onset of symptoms related to PANDAS (including TS). Finally, Lotan et al. (2014) extended these results by the identification of the serotonergic system as an additional mediator of the onset of PANDAS related symptoms. Specifically, beside replicating the presence of antibodies against D1 and D2 receptors, they observed peripheral antibodies against serotonin (5HT-2A and 5HT-2C) receptors in rats previously exposed to GABHS (Lotan et al., 2014). Furthermore, the active immunization of male Lewis rats resulted in a series of phenotypic abnormalities associated with compulsive behavior and motor impairments: increased grooming; impairments in food manipulation; and impairments in fine motor activity tested through walking on a narrow beam (Lotan et al., 2014). These observations are in line with pharmacological evidence indicating that several serotonergic agonists may constitute an effective treatment for the GABHS-dependent psychiatric symptoms (Swedo and Grant, 2005; Murphy et al., 2010). Additionally, these results parallel our study in which we showed that active streptococcal immunization throughout development may alter serotonergic transmission in the adult brain (Macrì et al., 2015). In the same study, Lotan et al. (2014) addressed whether the antibodies produced in response to GABHS were sufficient to induce an abnormal phenotype. To investigate this aspect, the authors performed a passive transfer experiment in which they injected purified IgG from immunized and control rats directly in the striatum of naïve rats (Lotan et al., 2014). In accordance with the predictions, microinfusion of IgG from immunized rats partially reproduced the phenotype of rats exposed to the direct immunization: impairments in food manipulation and in beam walking test (Lotan et al., 2014). Finally, immunoistochemical analysis of IgG deposits in striatum revealed the presence of IgG clusters in striatum of rats passively exposed to GABHS; moreover, the authors observed that these clusters co-localized with D1 and D2 receptors and with serotonin transporter (SERT; Lotan et al., 2014).

## Limitations of the studies and future perspectives

In the present manuscript, we aimed at describing animal models developed to investigate the link between streptococcus infections and the onset of autoimmune-mediated neuropsychiatric disorder. Within this framework, animal models developed using active immunization constitute a valid tool to investigate the etiological mechanisms of PANDAS and other related disorders, such as TS. Yet, these animal models present a series of limitations that need to be addressed in future experimental studies. Specifically, current experimental models are limited in terms of the timing of symptoms observation (prepubertal onset in humans in spite of the fact that abnormalities in rodents are generally addressed in already adult subjects) and in the limited exploitation of gene × environment interactions. In the following section, we discuss these limitations and propose an approach to overcome them in the future (these aspects are summarized in Table 2). As briefly mentioned, one of the core limitations is represented by the timing of the onset of PANDAS-like phenotype in preclinical models. PANDAS, as already discussed, are a series of streptococcal-related disorders that occur specifically in the pediatric population. Most of the PANDAS-related symptoms observed in animals (stereotypies, repetitive and perseverative behavior, impaired sensorimotor gating) have instead been addressed in late adolescent/adult individuals. Such limitation is predominantly related to technical constraints associated with the immunization protocol. In all the studies analyzed, the first immunization of a repeated protocol has been performed at four (Hoffman et al., 2004; Yaddanapudi et al., 2010; Macrì et al., 2015), five (Brimberg et al., 2012), or 6 weeks (Hoffman et al., 2004; Yaddanapudi et al., 2010) of age, corresponding, in rodents, to puberty and adolescence. Moreover, the subsequent injections (performed to simulate a repeated exposure to streptococcus) were always interspaced by 3 weeks. Thus, the consequences of the repeated exposure to streptococcus have been evaluated in fully adult mice. To assess the effects of streptococcus in younger individuals and come closer to the specific characteristic of the pediatric population, future studies shall entail an earlier timing of the primary injection, and much shorter intervals between boosts.

**Table 2:** **Experimental models of PANDAS and TS**.

**Pandas and TS**	**Experimental models of pandas and TS**	**Future perspectives**
**Chronic tic disorders and/or OCD^1^**	**OCD-like behaviors: increased repetitive behaviors, stereotypies, perseverative behavior and fine motor coordination^4−11^**	Extension of the behavioral phenotype to incorporate patterns analogous to tics
**Association with streptococcal infections^1^**	**Association with streptococcal infections through active immunization^7–11^ or passive transfer of sera from TS patients^4–6^**	Design of complex experimental protocols entailing a double hit hypothesis (e.g., stress during pregnancy x streptococcal immunization early in puberty)
**Motor hyperactivity^1^**	Motor hyperactivity	Prolongation of the analysis of motor behavior (e.g., continuous monitoring of behavior through automated systems)
**Prepubertal onset^1^**	Prepubertal onset	Earlier administration of streptococcal antigens
**Relapsing and remitting course of the disease^1^**	Relapsing and remitting course of the disease	–
**Impaired PPI^2^**	**Impaired PPI^9^**	–
*Autoantibodies developed following streptococcal infections, cross the BBB and bind specific target at the level of BG, causing morphological alteration in CNS*^3^	**IgG deposits in cerebellum, striatum, hippocampus and paraventricular area^7, 8^ inflammation in rostral diencephalon^9^**	Investigation of the role of microglia and astrocytes in the autoimmune sequelae in preclinical models^12^

*Bold, Scientific evidence; Italics, hypothesis; Normal text, Clinical evidence not reproduced in animal models*.

*Numbers indicate the references in which the corresponding information has been described. (1) Swedo et al., 1998; (2) Swerdlow et al., 2001b; (3) Martino et al., 2009; (4) Hallett et al., 2000; (5) Taylor et al., 2002; (6) Yeh et al., 2012; (7) Hoffman et al., 2004; (8) Yaddanapudi et al., 2010; (9) Macrì et al., 2015; (10) Brimberg et al., 2012; (11) Lotan et al., 2014; (12) Benedek et al., 2016*.

### Animal models and gene × environment interactions

With particular attention to TS, the utility of autoimmune models should be extended to investigate the role gene × environment interactions. Considering the multifactorial and complex etiology of TS (entailing also genetic vulnerability), several experimental models leveraged the use of genetically-engineered animals. For example, SLITRK knockout (ko) mice, have been developed following the identification of mutations in SLITRK genes in TS patients (Katayama et al., 2010). Although Slitrk1-deficient mice possess a great degree of construct validity, this model does not resemble TS symptomatology, thereby possessing a limited degree of face validity. In particular, Sltrk1 ko mice did not show tic-like symptoms or neurochemical abnormalities typical of TS. Other genetic models have been developed based on the link between TS and glutamatergic hyperactivity inside the CSTC. In particular, Nordstrom and colleagues developed a D1 receptor transgenic animal model (D1CT-7), expressing hyperactivity in two groups of neural populations located (expressing D1 receptors) in the insular and piriform cortices and in the amygdala (Nordstrom and Burton, 2002). These mice exhibit features that are analogous to the tics observed in TS patients (very brief isolated head or body *jerk* or *shake*); moreover, these symptoms are alleviated by the administration of drugs that have analogous effects in humans. Despite the good degree of face and predictive validity of this model, D1CT-7 mice exhibit also features that are not typical of TS (see Macrì et al., 2013 for a detailed review). Recently, Castellan Baldan et al. (2014) developed an animal model resting upon the observation that the *hdc* gene may be involved in TS (Ercan-Sencicek et al., 2010). Within this framework, the authors developed a line of mice (*hdc* ko) lacking histidine decarboxylase, which is necessary to synthesize histamine (Castellan Baldan et al., 2014). These mice exhibited a significant increase in behavioral stereotypies after D-amphetamine injection and a deficit in sensorimotor gating, reflected in impairments in PPI. Haloperidol pretreatment mitigated behavioral stereotypies. Moreover, while in the brain of wild type mice HA concentration increased during the dark-active phase of the diurnal cycle (Haas et al., 2008) HA concentration was significantly reduced in left hypothalamus, striatum and right neocortex of ko mice (Castellan Baldan et al., 2014). Although daytime striatal DA concentration did not differ between genotypes, *hdc* ko mice showed a significant increase in DA concentration during the active phase of the diurnal cycle, when HA was increased in wild type mice (Castellan Baldan et al., 2014). This result further supports the hypothesis that HA negatively regulates DA (Haas et al., 2008). Finally, ko mice showed a reduction of D2 + D3 receptor density in striatum, and an increase of D2 + D3 receptor density in substantia nigra, suggesting a cellular response to the persistent elevation of DA (Stanwood et al., 2000). Thus, in the light of the theoretical framework in which it has been developed (clinical evidence indicating the importance of HA in TS; Ercan-Sencicek et al., 2010), the phenotypic alterations observed (motor abnormalities and impaired sensorimotor gating), and the pharmacological efficacy of haloperidol (one of the treatments of choice in TS, Bornstein et al., 1990), this experimental model seems to possess an elevated degree of construct, face and predictive validity.

Given the limitations of both autoimmune and genetic models, future attempts should be focused on combining some of the models previously described, aiming at investigating gene × environment interactions.

### Neonatal environmental factors and individual vulnerability to PANDAS

Future efforts should be focused also on investigating the possibility that neonatal environmental factors may calibrate, and eventually suppress, individual vulnerability to PANDAS. Several studies reported that precocious experiences affect the individual resilience toward future challenges (Heim et al., 2004, 2008; Lyons and Macrì, 2011). In particular, while traumatic precocious experiences result in increased individual vulnerability to future challenges (Heim et al., 2004, 2008), stimulating early conditions have been proposed to favor individual resilience (Lyons and Macrì, 2011). The immune system is particularly sensitive to early experiences (Roque et al., 2014). For example, several studies conducted in rodents showed that maternal separation (daily 3–6 h mother-offspring separations during the first 2 weeks of life) or exposure to early physiological stressors result in increased susceptibility toward viral infections (Meagher et al., 2010) or vulnerability toward autoimmune phenomena (Bakker et al., 2000). Individual reactivity to immune challenge has been proposed to depend on the functionality of hypothalamic-pituitary-adrenocortical (HPA) axis (Bakker et al., 2000; Meagher et al., 2010). In particular, the differential response to immune challenge depends on the modulation of the functionality of immune system exerted by elevations in levels of corticosterone (Laban et al., 1995). Several studies showed that circulating corticosteroids have a direct effect on T-cell, suppressing immune responses (Wick et al., 1993). Thus, it is tenable to propose that the modulation of corticosterone reactivity through experimental stressors, may calibrate individual susceptibility toward phenomena that relate to immunity (immune and autoimmune phenomena). Within this framework, Levine and Saltzman (1987) reported that experimental stressors favoring an upregulation of the HPA-axis alter individual vulnerability to EAE (Levine and Saltzman, 1987). Moreover, Levine and colleagues showed that stress reduces (Levine et al., 1962a) and adrenalectomy enhances (Levine et al., 1962b) vulnerability to EAE. Thus, a persistent upregulation of HPA axis induced by neonatal stressor may consistently prevent some of the consequences of experimental models of autoimmunity, such as PANDAS. Beside analyzing the role of environmental factors in modulating the functionality of the immune system, future studies shall thoroughly detail which portions of the immune system are involved in the autoimmune sequelae (Benedek et al., 2016). An interesting target to be contemplated in future studies is the activation of microglia and the role exerted by astrocytes (Benedek et al., 2016; Lécuyer et al., 2016). These targets appear particularly relevant whereby their involvement has already been demonstrated in experimental models of autoimmunity (Correale, 2014; Shemer and Jung, 2015; Benedek et al., 2016; Lécuyer et al., 2016). While these studies addressed the role of astrocytes and microglia in experimental models of MS, it may be important to evaluate whether these outcomes translate to experimental models of PANDAS or TS. This need is also corroborated by clinical evidence indicating that microglia can be activated in TS and PANDAS patients (Kumar et al., 2015), see below for a detailed description.

### Peripheral autoantibodies as diagnostic biomarker in TS

Finally, we emphasize the value of the detection of peripheral autoantibodies as a reliable method potentially aiding the diagnosis of neurological disorders. The search of biological markers measurable and detectable using non-invasive approaches constitutes an important tool in the diagnosis of these diseases (Damoiseaux et al., 2015). With particular attention to autoimmune disorders, the measurement of autoantibodies has been proposed as a valuable tool not only in the diagnosis, but also in the prediction and in the prognosis of autoimmune diseases (Harel and Shoenfeld, 2006; Shepshelovich and Shoenfeld, 2006; Bizzaro et al., 2007; see Damoiseaux et al., 2015 for a detailed review). Within this framework, several peripheral autoantibodies emerged as clinically relevant in several neurological and neuropsychiatric disorders, such as multiple sclerosis (Comabella and Montalban, 2014), limbic encephalitis (Beck et al., 2009; Schlumberger et al., 2014), myasthenia gravis (Verschuuren et al., 2013), or ADHD (Giana et al., 2015). However, the debate on the efficacy of serum autoantibodies as diagnostic markers is still open. For example Höftberger (2015) reported that in the case of autoimmune encephalitis (AIE), serum did not contain antineural antibodies in the 14% of patients, while autoantibodies were always detected in patients' cerebrospinal fluid (CSF). Thus, the absence of autoantibodies in serum may not be sufficient to exclude AIE (Höftberger, 2015). With respect to TS, as already discussed (see Introduction), several clinical studies reported the presence of anti-BG antibodies in sera of TS patients (Rizzo et al., 2006; Martino et al., 2011). Moreover, the injections of TS sera (containing autoantibodies) directly into rodents' striatum, result, in some cases, in behavioral and neurochemical alterations that partially resemble PANDAS symptomatology (Hallett et al., 2000; Yeh et al., 2012). In addition, autoantibodies in TS sera seem to induce PANDAS-like behavioral phenotypes in a concentration-dependent manner (high or low level of autoantibodies titers, see Taylor et al., 2002). These results seem to support the hypothesis that anti-BG antibodies may constitute a valid biomarker in the diagnosis of some cases of TS. However, results reported in subsequent studies, where the passive transfer of TS sera in rodents' striatum failed to induce PANDAS-like phenotypes (Loiselle et al., 2004; Ben-Pazi et al., 2012), suggest that additional studies are necessary to investigate the diagnostic value of the detection of peripheral BG-antigens in TS. Further efforts may be focused, for example, on the standardization of the assays used to quantify antineural antibodies in sera (Jacobs et al., 2015). In particular, further studies should be focused on investigating the use of immunohistochemistry as a method for the detection of anti-neuronal autoantibodies in CNS disorders. Hachiya et al. (2013) showed, in a recent study, that immunohistochemistry may constitute a reliable method for the detection of autoantibodies in serum of patients affected by CNS disorders associated with GABHS infections (Hachiya et al., 2013). In particular, they assessed immunoreactivity of sera (obtained during the acute phase of disease or during remission or convalescence) from patients affected by three CNS disorders linked to GABHS infections (acute disseminated encephalomyelitis, PANDAS and subacute encephalitis). The authors performed immunohistochemistry on brain sections of hippocampus, basal ganglia, cerebellar cortex and midbrain obtained from male controls (aged 5 and 9 years) that did not present CNS alterations. Sera obtained from patients affected by acute disseminated encephalomyelitis and PANDAS showed immunoreactivity in globus pallidus neurons, while sera obtained from patients affected by subacute encephalitis showed immunoreactivity in the extra pyramidal cell layers in the temporal cortex. Conversely, sera obtained during remission or convalescence did not show immunoreactivity (Hachiya et al., 2013). With particular attention to PANDAS and TS, future efforts should be focused, for example, on analyzing immunoreactivity of sera from patients toward dopamine D2 receptors. In the context of the identification of immune-related diagnostic biomarkers, Kumar et al. (2015) recently evaluated neuroinflammation in TS and PANDAS children. Specifically, the authors performed a Positron Emission Tomographic (PET) study to identify markers of activated microglia (Kumar et al., 2015). Activated microglia has been proposed to constitute a valid indicator of the presence of neuroinflammation (Kreutzberg, 1996). To address this aspect, the authors exploited the capacity of activated microglia to express the translocator protein receptor (TSPO). TSPO, in turn, can be selectively identified through the radioactive tracer ^11^C-[R]-PK11195 (PK) (Cagnin et al., 2007). Using this approach, the authors analyzed neuroinflammation in basal ganglia and thalamus and observed increased binding potential in bilateral caudate and bilateral lentiform nucleus in PANDAS patients. TS children exhibited neuroinflammation in bilateral lentiform nucleus only, suggesting possible neuroanatomical differences between PANDAS and TS diseases (Kumar et al., 2015). Thus, the monitoring of neuroinflammation through PET may constitute a potential method to clarify pathophysiological mechanisms in TS and PANDAS.

## Author contributions

CS and SM wrote the first draft of the manuscript; CS, SM, and GL worked on the subsequent versions of the manuscript.

### Conflict of interest statement

The authors declare that the research was conducted in the absence of any commercial or financial relationships that could be construed as a potential conflict of interest.

## References

[B1] Abelson J. F. Kwan K. Y. O'Roak B. J. Baek D. Y. Stillman A. A. Morgan T. M.. (2005). Sequence variants in SLITRK1 are associated with Tourette's syndrome. Science 310, 317–320. 10.1126/science.1116502 16224024

[B2] Acharjee S. Nayani N. Tsutsui M. Hill M. N. Ousman S. S. Pittman Q. J. (2013). Altered cognitive-emotional behavior in early experimental autoimmune encephalitis–cytokine and hormonal correlates. Brain Behav. Immun. 33, 164–172. 10.1016/j.bbi.2013.07.003 23886782

[B3] Adriani W. Koot S. Columba-Cabezas S. Romano E. Travaglini D. van den Bos R.. (2012). Immunization with DAT fragments is associated with long-term striatal impairment, hyperactivity and reduced cognitive flexibility in mice. Behav. Brain Funct. 8:54. 10.1186/1744-9081-8-54 23192105 PMC3537576

[B4] Ahmari S. E. Risbrough V. B. Geyer M. A. Simpson H. B. (2012). Impaired sensorimotor gating in unmedicated adults with obsessive-compulsive disorder. Neuropsychopharmacology 37, 1216–1223. 10.1038/npp.2011.308 22218093 PMC3306882

[B5] Almutairi M. M. Gong C. Xu Y. G. Chang Y. Shi H. (2016). Factors controlling permeability of the blood-brain barrier. Cell. Mol. Life Sci. 73, 57–77. 10.1007/s00018-015-2050-8 26403789 PMC11108286

[B6] Amor S. Baker D. Groome N. Turk J. L. (1993). Identification of a major encephalitogenic epitope of proteolipid protein (residues 56–70) for the induction of experimental allergic encephalomyelitis in Biozzi AB/H and nonobese diabetic mice. J. Immunol. 150, 5666–5672. 7685799

[B7] Amor S. Groome N. Linington C. Morris M. M. Dornmair K. Gardinier M. V.. (1994). Identification of epitopes of myelin oligodendrocyte glycoprotein for the induction of experimental allergic encephalomyelitis in SJL and Biozzi AB/H mice. J. Immunol. 153, 4349–4356. 7525700

[B8] Amor S. O'Neill J. K. Morris M. M. Smith R. M. Wraith D. C. Groome N.. (1996). Encephalitogenic epitopes of myelin basic protein, proteolipid protein, myelin oligodendrocyte glycoprotein for experimental allergic encephalomyelitis induction in Biozzi ABH (H-2Ag7) mice share an amino acid motif. J. Immunol. 156, 3000–3008. 8609422

[B9] APA (2013). Diagnostic and Statistical Manual of Mental Disorders. Arlington, VA: American Psychiatric Association.

[B10] Aruga J. Mikoshiba K. (2003). Identification and characterization of Slitrk, a novel neuronal transmembrane protein family controlling neurite outgrowth. Mol. Cell. Neurosci. 24, 117–129. 10.1016/S1044-7431(03)00129-5 14550773

[B11] Asano N. M. Coriolano M. Asano B. J. Lins O. G. (2013). Psychiatric comorbidities in patients with systemic lupus erythematosus: a systematic review of the last 10 years. Rev. Bras. Reumatol. 53, 431–437. 10.1016/S2255-5021(13)70114-7 24316900

[B12] Bakker J. M. Kavelaars A. Kamphuis P. J. Cobelens P. M. van Vugt H. H. van Bel F.. (2000). Neonatal dexamethasone treatment increases susceptibility to experimental autoimmune disease in adult rats. J. Immunol. 165, 5932–5937. 10.4049/jimmunol.165.10.5932 11067955

[B13] Baldan Ramsey L. C. Xu M. Wood N. Pittenger C. (2011). Lesions of the dorsomedial striatum disrupt prepulse inhibition. Neuroscience 180, 222–228. 10.1016/j.neuroscience.2011.01.041 21315809 PMC3070827

[B14] Beck L. H. Jr. Bonegio R. G. Lambeau G. Beck D. M. Powell D. W. Cummins T. D.. (2009). M-type phospholipase A2 receptor as target antigen in idiopathic membranous nephropathy. N. Engl. J. Med. 361, 11–21. 10.1056/NEJMoa0810457 19571279 PMC2762083

[B15] Benedek G. Zhang J. Bodhankar S. Nguyen H. Kent G. Jordan K.. (2016). Estrogen induces multiple regulatory B cell subtypes and promotes M2 microglia and neuroprotection during experimental autoimmune encephalomyelitis. J. Neuroimmunol. 293, 45–53. 10.1016/j.jneuroim.2016.02.009 27049561 PMC4824954

[B16] Ben-Pazi H. Sadan O. Offen D. (2012). Striatal microinjection of Sydenham chorea antibodies: using a rat model to examine the dopamine hypothesis. J. Mol. Neurosci. 46, 162–166. 10.1007/s12031-011-9559-6 21647711

[B17] Berardelli A. Currà A. Fabbrini G. Gilio F. Manfredi M. (2003). Pathophysiology of tics and Tourette syndrome. J. Neurol. 250, 781–787. 10.1007/s00415-003-1102-4 12883917

[B18] Bizzaro N. Tonutti E. Visentini D. Alessio M. G. Platzgummer S. Morozzi G.. (2007). Antibodies to the lens and cornea in anti-DFS70-positive subjects. Ann. N.Y. Acad. Sci. 1107, 174–183. 10.1196/annals.1381.019 17804545

[B19] Boghosian-Sell L. Comings D. E. Overhauser J. (1996). Tourette syndrome in a pedigree with a 7;18 translocation: identification of a YAC spanning the translocation breakpoint at 18q22.3. Am. J. Hum. Genet. 59, 999–1005. 8900226 PMC1914824

[B20] Borchers A. T. Aoki C. A. Naguwa S. M. Keen C. L. Shoenfeld Y. Gershwin M. E. (2005). Neuropsychiatric features of systemic lupus erythematosus. Autoimmun. Rev. 4, 329–344. 10.1016/j.autrev.2005.01.008 16081024

[B21] Bornstein R. A. Stefl M. E. Hammond L. (1990). A survey of Tourette syndrome patients and their families: the 1987 Ohio Tourette Survey. J. Neuropsychiatry Clin. Neurosci. 2, 275–281. 2136086 10.1176/jnp.2.3.275

[B22] Bos-Veneman N. G. Olieman R. Tobiasova Z. Hoekstra P. J. Katsovich L. Bothwell A. L.. (2011). Altered immunoglobulin profiles in children with Tourette syndrome. Brain Behav. Immun. 25, 532–538. 10.1016/j.bbi.2010.12.003 21156204 PMC3056238

[B23] Brilot F. Merheb V. Ding A. Murphy T. Dale R. C. (2011). Antibody binding to neuronal surface in Sydenham chorea, but not in PANDAS or Tourette syndrome. Neurology 76, 1508–1513. 10.1212/WNL.0b013e3182181090 21411742 PMC3087465

[B24] Brimberg L. Benhar I. Mascaro-Blanco A. Alvarez K. Lotan D. Winter C.. (2012). Behavioral, pharmacological, and immunological abnormalities after streptococcal exposure: a novel rat model of Sydenham chorea and related neuropsychiatric disorders. Neuropsychopharmacology 37, 2076–2087. 10.1038/npp.2012.56 22534626 PMC3398718

[B25] Bronson S. L. Bale T. L. (2016). The Placenta as a mediator of stress effects on neurodevelopmental reprogramming. Neuropsychopharmacology 41, 207–218. 10.1038/npp.2015.231 26250599 PMC4677129

[B26] Cagnin A. Kassiou M. Meikle S. R. Banati R. B. (2007). Positron emission tomography imaging of neuroinflammation. Neurotherapeutics 4, 443–452. 10.1016/j.nurt.2007.04.006 17599710 PMC7479716

[B27] Capone F. Adriani W. Shumilina M. Izykenova G. Granstrem O. Dambinova S.. (2008). Autoantibodies against opioid or glutamate receptors are associated with changes in morphine reward and physical dependence in mice. Psychopharmacology 197, 535–548. 10.1007/s00213-007-1062-y 18265961

[B28] Cardona F. Orefici G. (2001). Group A streptococcal infections and tic disorders in an Italian pediatric population. J. Pediatr. 138, 71–75. 10.1067/mpd.2001.110325 11148515

[B29] Cardoso F. Vargas A. P. Oliveira L. D. Guerra A. A. Amaral S. V. (1999). Persistent Sydenham's chorea. Mov. Disord. 14, 805–807. 10495042 10.1002/1531-8257(199909)14:5<805::aid-mds1013>3.0.co;2-p

[B30] Castellan Baldan L. Williams K. A. Gallezot J. D. Pogorelov V. Rapanelli M. Crowley M.. (2014). Histidine decarboxylase deficiency causes tourette syndrome: parallel findings in humans and mice. Neuron 81, 77–90. 10.1016/j.neuron.2013.10.052 24411733 PMC3894588

[B31] Castellanos F. X. Fine E. J. Kaysen D. Marsh W. L. Rapoport J. L. Hallett M. (1996). Sensorimotor gating in boys with Tourette's syndrome and ADHD: preliminary results. Biol. Psychiatry 39, 33–41. 10.1016/0006-3223(95)00101-8 8719124

[B32] Cieza A. Anczewska M. Ayuso-Mateos J. L. Baker M. Bickenbach J. Chatterji S.. (2015). Understanding the impact of brain disorders: towards a ‘horizontal epidemiology’ of psychosocial difficulties and their determinants. PLoS ONE 10:e0136271. 10.1371/journal.pone.0136271 26352911 PMC4564202

[B33] Coenen M. Cabello M. Umlauf S. Ayuso-Mateos J. L. Anczewska M. Tourunen J.. (2016). Psychosocial difficulties from the perspective of persons with neuropsychiatric disorders. Disabil. Rehabil. 38, 1134–1145. 10.3109/09638288.2015.1074729 26289372

[B34] Comabella M. Montalban X. (2014). Body fluid biomarkers in multiple sclerosis. Lancet Neurol. 13, 113–126. 10.1016/S1474-4422(13)70233-3 24331797

[B35] Correale J. (2014). The role of microglial activation in disease progression. Mult. Scler. 20, 1288–1295. 10.1177/1352458514533230 24812046

[B36] Cutforth T. DeMille M. M. Agalliu I. Agalliu D. (2016). CNS autoimmune disease after infections: animal models, cellular mechanisms and genetic factors. Future Neurol. 11, 63–76. 10.2217/fnl.16.4 27110222 PMC4839655

[B37] Dale R. C. Church A. J. Candler P. M. Chapman M. Martino D. Giovannoni G. (2006). Serum autoantibodies do not differentiate PANDAS and Tourette syndrome from controls. Neurology 66:1612. 10.1212/01.wnl.0000226832.36908.4c 16717245

[B38] Dalsgaard S. Waltoft B. L. Leckman J. F. Mortensen P. B. (2015). Maternal history of autoimmune disease and later development of tourette syndrome in offspring. J. Am. Acad. Child Adolesc. Psychiatry 54, 495–501.e1. 10.1016/j.jaac.2015.03.008 26004665

[B39] Damoiseaux J. Andrade L. E. Fritzler M. J. Shoenfeld Y. (2015). Autoantibodies 2015: from diagnostic biomarkers toward prediction, prognosis and prevention. Autoimmun. Rev. 14, 555–563. 10.1016/j.autrev.2015.01.017 25661979

[B40] Davison K. (2012). Autoimmunity in psychiatry. Br. J. Psychiatry 200, 353–355. 10.1192/bjp.bp.111.104471 22550326

[B41] Deacon R. M. Rawlins J. N. (2006). T-maze alternation in the rodent. Nat. Protoc. 1, 7–12. 10.1038/nprot.2006.2 17406205

[B42] Deng H. Gao K. Jankovic J. (2012). The genetics of Tourette syndrome. Nat. Rev. Neurol. 8, 203–213. 10.1038/nrneurol.2012.26 22410579

[B43] Dileepan T. Linehan J. L. Moon J. J. Pepper M. Jenkins M. K. Cleary P. P. (2011). Robust antigen specific th17 T cell response to group A Streptococcus is dependent on IL-6 and intranasal route of infection. PLoS Pathog. 7:e1002252. 10.1371/journal.ppat.1002252 21966268 PMC3178561

[B44] Dileepan T. Smith E. D. Knowland D. Hsu M. Platt M. Bittner-Eddy P.. (2016). Group A Streptococcus intranasal infection promotes CNS infiltration by streptococcal-specific Th17 cells. J. Clin. Invest. 126, 303–317. 10.1172/JCI80792 26657857 PMC4701547

[B45] Ebrahimi A. Pochet R. Roger M. (1992). Topographical organization of the projections from physiologically identified areas of the motor cortex to the striatum in the rat. Neurosci. Res. 14, 39–60. 1380687 10.1016/s0168-0102(05)80005-7

[B46] Ercan-Sencicek A. G. Stillman A. A. Ghosh A. K. Bilguvar K. O'Roak B. J. Mason C. E.. (2010). L-histidine decarboxylase and Tourette's syndrome. N. Engl. J. Med. 362, 1901–1908. 10.1056/NEJMoa0907006 20445167 PMC2894694

[B47] Fanouriakis A. Boumpas D. T. Bertsias G. K. (2013). Pathogenesis and treatment of CNS lupus. Curr. Opin. Rheumatol. 25, 577–583. 10.1097/BOR.0b013e328363eaf1 23836074

[B48] Fernandez T. V. Sanders S. J. Yurkiewicz I. R. Ercan-Sencicek A. G. Kim Y. S. Fishman D. O.. (2012). Rare copy number variants in tourette syndrome disrupt genes in histaminergic pathways and overlap with autism. Biol. Psychiatry 71, 392–402. 10.1016/j.biopsych.2011.09.034 22169095 PMC3282144

[B49] Ganor Y. Freilinger M. Dulac O. Levite M. (2005a). Monozygotic twins discordant for epilepsy differ in the levels of potentially pathogenic autoantibodies and cytokines. Autoimmunity 38, 139–150. 10.1080/08916930500100825 16040334

[B50] Ganor Y. Goldberg-Stern H. Amrom D. Lerman-Sagie T. Teichberg V. I. Pelled D.. (2004). Autoimmune epilepsy: some epilepsy patients harbor autoantibodies to glutamate receptors and dsDNA on both sides of the blood-brain barrier, which may kill neurons and decrease in brain fluids after hemispherotomy. Clin. Dev. Immunol. 11, 241–252. 10.1080/17402520400001736 15559370 PMC2486323

[B51] Ganor Y. Goldberg-Stern H. Blank M. Shoenfeld Y. Dobrynina L. A. Kalashnikova L.. (2005c). Antibodies to glutamate receptor subtype 3 (GluR3) are found in some patients suffering from epilepsy as the main disease, but not in patients whose epilepsy accompanies antiphospholipid syndrome or Sneddon's syndrome. Autoimmunity 38, 417–424. 10.1080/08916930500246339 16278146

[B52] Ganor Y. Goldberg-Stern H. Cohen R. Teichberg V. Levite M. (2014). Glutamate receptor antibodies directed against AMPA receptors subunit 3 peptide B (GluR3B) can be produced in DBA/2J mice, lower seizure threshold and induce abnormal behavior. Psychoneuroendocrinology 42, 106–117. 10.1016/j.psyneuen.2014.01.005 24636507

[B53] Ganor Y. Goldberg-Stern H. Lerman-Sagie T. Teichberg V. I. Levite M. (2005b). Autoimmune epilepsy: distinct subpopulations of epilepsy patients harbor serum autoantibodies to either glutamate/AMPA receptor GluR3, glutamate/NMDA receptor subunit NR2A or double-stranded DNA. Epilepsy Res. 65, 11–22. 10.1016/j.eplepsyres.2005.03.011 15978777

[B54] Giana G. Romano E. Porfirio M. C. D'Ambrosio R. Giovinazzo S. Troianiello M.. (2015). Detection of auto-antibodies to DAT in the serum: interactions with DAT genotype and psycho-stimulant therapy for ADHD. J. Neuroimmunol. 278, 212–222. 10.1016/j.jneuroim.2014.11.008 25468771

[B55] Goldberg-Stern H. Ganor Y. Cohen R. Pollak L. Teichberg V. Levite M. (2014). Glutamate receptor antibodies directed against AMPA receptors subunit 3 peptide B (GluR3B) associate with some cognitive/psychiatric/behavioral abnormalities in epilepsy patients. Psychoneuroendocrinology 40, 221–231. 10.1016/j.psyneuen.2013.11.007 24485494

[B56] Haas H. L. Sergeeva O. A. Selbach O. (2008). Histamine in the nervous system. Physiol. Rev. 88, 1183–1241. 10.1152/physrev.00043.2007 18626069

[B57] Hachiya Y. Miyata R. Tanuma N. Hongou K. Tanaka K. Shimoda K.. (2013). Autoimmune neurological disorders associated with group-A beta-hemolytic streptococcal infection. Brain Dev. 35, 670–674. 10.1016/j.braindev.2012.10.003 23142103

[B58] Hallett J. J. Harling-Berg C. J. Knopf P. M. Stopa E. G. Kiessling L. S. (2000). Anti-striatal antibodies in Tourette syndrome cause neuronal dysfunction. J. Neuroimmunol. 111, 195–202. 10.1016/S0165-5728(00)00320-9 11063838

[B59] Harel M. Shoenfeld Y. (2006). Predicting and preventing autoimmunity, myth or reality? Ann. N.Y. Acad. Sci. 1069, 322–345. 10.1196/annals.1351.031 16855160

[B60] Hartley S. McArthur M. Coenen M. Cabello M. Covelli V. Roszczynska-Michta J.. (2014). Narratives reflecting the lived experiences of people with brain disorders: common psychosocial difficulties and determinants. PLoS ONE 9:e96890. 10.1371/journal.pone.0096890 24805128 PMC4013080

[B61] Heim C. Newport D. J. Mletzko T. Miller A. H. Nemeroff C. B. (2008). The link between childhood trauma and depression: insights from HPA axis studies in humans. Psychoneuroendocrinology 33, 693–710. 10.1016/j.psyneuen.2008.03.008 18602762

[B62] Heim C. Plotsky P. M. Nemeroff C. B. (2004). Importance of studying the contributions of early adverse experience to neurobiological findings in depression. Neuropsychopharmacology 29, 641–648. 10.1038/sj.npp.1300397 15034558

[B63] Hoekstra P. J. Dietrich A. Edwards M. J. Elamin I. Martino D. (2013). Environmental factors in Tourette syndrome. Neurosci. Biobehav. Rev. 37, 1040–1049. 10.1016/j.neubiorev.2012.10.010 23092654

[B64] Hoenig K. Hochrein A. Quednow B. B. Maier W. Wagner M. (2005). Impaired prepulse inhibition of acoustic startle in obsessive-compulsive disorder. Biol. Psychiatry 57, 1153–1158. 10.1016/j.biopsych.2005.01.040 15866555

[B65] Hoffman K. L. Hornig M. Yaddanapudi K. Jabado O. Lipkin W. I. (2004). A murine model for neuropsychiatric disorders associated with group A beta-hemolytic streptococcal infection. J. Neurosci. 24, 1780–1791. 10.1523/JNEUROSCI.0887-03.2004 14973249 PMC6730451

[B66] Höftberger R. (2015). Neuroimmunology: an expanding frontier in autoimmunity. Front. Immunol. 6:206. 10.3389/fimmu.2015.00206 25972873 PMC4413830

[B67] Hornig M. (2013). The role of microbes and autoimmunity in the pathogenesis of neuropsychiatric illness. Curr. Opin. Rheumatol. 25, 488–795. 10.1097/BOR.0b013e32836208de 23656715

[B68] Hornig M. Lipkin W. I. (2013). Immune-mediated animal models of Tourette syndrome. Neurosci. Biobehav. Rev. 37, 1120–1138. 10.1016/j.neubiorev.2013.01.007 23313649 PMC4054816

[B69] Huerta P. T. Kowal C. DeGiorgio L. A. Volpe B. T. Diamond B. (2006). Immunity and behavior: antibodies alter emotion. Proc. Natl. Acad. Sci. U.S.A. 103, 678–683. 10.1073/pnas.0510055103 16407105 PMC1334673

[B70] Huppert J. Closhen D. Croxford A. White R. Kulig P. Pietrowski E.. (2010). Cellular mechanisms of IL-17-induced blood-brain barrier disruption. FASEB J. 24, 1023–1034. 10.1096/fj.09-141978 19940258

[B71] Hyman S. E. (2008). A glimmer of light for neuropsychiatric disorders. Nature 455, 890–893. 10.1038/nature07454 18923510

[B72] Irani S. R. Gelfand J. M. Al-Diwani A. Vincent A. (2014). Cell-surface central nervous system autoantibodies: clinical relevance and emerging paradigms. Ann. Neurol. 76, 168–184. 10.1002/ana.24200 24930434 PMC4141019

[B73] Irwin J. Tombaugh T. N. Zacharko R. M. Anisman H. (1983). Alteration of exploration and the response to food associated cues after treatment with pimozide. Pharmacol. Biochem. Behav. 18, 235–246. 6835981 10.1016/0091-3057(83)90369-6

[B74] Jacobs J. F. van der Molen R. G. Bossuyt X. Damoiseaux J. (2015). Antigen excess in modern immunoassays: to anticipate on the unexpected. Autoimmun. Rev. 14, 160–167. 10.1016/j.autrev.2014.10.018 25461469

[B75] Jaffard R. Mocaer E. Poignant J. C. Micheau J. Marighetto A. Meunier M.. (1991). Effects of tianeptine on spontaneous alternation, simple and concurrent spatial discrimination learning and on alcohol-induced alternation deficits in mice. Behav. Pharmacol. 2, 37–46. 11224046

[B76] John C. C. Carabin H. Montano S. M. Bangirana P. Zunt J. R. Peterson P. K. (2015). Global research priorities for infections that affect the nervous system. Nature 527, S178–S186. 10.1038/nature16033 26580325 PMC4697933

[B77] Kajiwara Y. Buxbaum J. D. Grice D. E. (2009). SLITRK1 binds 14-3-3 and regulates neurite outgrowth in a phosphorylation-dependent manner. Biol. Psychiatry 66, 918–925. 10.1016/j.biopsych.2009.05.033 19640509

[B78] Kaltwasser M. T. (1990). Acoustic signaling in the black rat (*Rattus rattus*). J. Comp. Psychol. 104, 227–232. 2225759 10.1037/0735-7036.104.3.227

[B79] Kalueff A. V. Stewart A. M. Song C. Berridge K. C. Graybiel A. M. Fentress J. C. (2016). Neurobiology of rodent self-grooming and its value for translational neuroscience. Nat. Rev. Neurosci. 17, 45–59. 10.1038/nrn.2015.8 26675822 PMC4840777

[B80] Katayama K. Yamada K. Ornthanalai V. G. Inoue T. Ota M. Murphy N. P.. (2010). Slitrk1-deficient mice display elevated anxiety-like behavior and noradrenergic abnormalities. Mol. Psychiatry 15, 177–184. 10.1038/mp.2008.97 18794888

[B81] Kawikova I. Leckman J. F. Kronig H. Katsovich L. Bessen D. E. Ghebremichael M.. (2007). Decreased numbers of regulatory T cells suggest impaired immune tolerance in children with tourette syndrome: a preliminary study. Biol. Psychiatry 61, 273–278. 10.1016/j.biopsych.2006.06.012 16996487

[B82] Kebir H. Kreymborg K. Ifergan I. Dodelet-Devillers A. Cayrol R. Bernard M.. (2007). Human TH17 lymphocytes promote blood-brain barrier disruption and central nervous system inflammation. Nat. Med. 13, 1173–1175. 10.1038/nm1651 17828272 PMC5114125

[B83] Kipp M. van der Star B. Vogel D. Y. Puentes F. van der Valk P. Baker D.. (2012). Experimental *in vivo* and *in vitro* models of multiple sclerosis: EAE and beyond. Mult. Scler. Relat. Disord. 1, 15–28. 10.1016/j.msard.2011.09.002 25876447

[B84] Korngold R. Feldman A. Rorke L. B. Lublin F. D. Doherty P. C. (1986). Acute experimental allergic encephalomyelitis in radiation bone marrow chimeras between high and low susceptible strains of mice. Immunogenetics 24, 309–315. 3781573 10.1007/BF00395536

[B85] Kowal C. Diamond B. (2012). Aspects of CNS lupus: mouse models of anti-NMDA receptor antibody mediated reactivity. Methods Mol. Biol. 900, 181–206. 10.1007/978-1-60761-720-4_9 22933070

[B86] Kowal C. DeGiorgio L. A. Nakaoka T. Hetherington H. Huerta P. T. Diamond B.. (2004). Cognition and immunity; antibody impairs memory. Immunity 21, 179–188. 10.1016/j.immuni.2004.07.011 15308099

[B87] Kreutzberg G. W. (1996). Microglia: a sensor for pathological events in the CNS. Trends Neurosci. 19, 312–318. 8843599 10.1016/0166-2236(96)10049-7

[B88] Kumar A. Williams M. T. Chugani H. T. (2015). Evaluation of basal ganglia and thalamic inflammation in children with pediatric autoimmune neuropsychiatric disorders associated with streptococcal infection and tourette syndrome: a positron emission tomographic (PET) study using 11C-[R]-PK11195. J. Child Neurol. 30, 749–756. 10.1177/0883073814543303 25117419

[B89] Laban O. Dimitrijevic M. von Hoersten S. Markovic B. M. Jankovic B. D. (1995). Experimental allergic encephalomyelitis in adult DA rats subjected to neonatal handling or gentling. Brain Res. 676, 133–140. 7540932 10.1016/0006-8993(95)00106-z

[B90] Lalonde R. (2002). The neurobiological basis of spontaneous alternation. Neurosci. Biobehav. Rev. 26, 91–104. 10.1016/S0149-7634(01)00041-0 11835987

[B91] Landau Y. E. Steinberg T. Richmand B. Leckman J. F. Apter A. (2012). Involvement of immunologic and biochemical mechanisms in the pathogenesis of Tourette's syndrome. J. Neural Transm. 119, 621–626. 10.1007/s00702-011-0739-x 22139323 PMC3936959

[B92] Leckman J. F. Peterson B. S. (1993). The pathogenesis of Tourette's syndrome: epigenetic factors active in early CNS development. Biol. Psychiatry 34, 425–427. 8268326 10.1016/0006-3223(93)90232-3

[B93] Leckman J. F. Bloch M. H. Smith M. E. Larabi D. Hampson M. (2010). Neurobiological substrates of Tourette's disorder. J. Child Adolesc. Psychopharmacol. 20, 237–247. 10.1089/cap.2009.0118 20807062 PMC2958453

[B94] Leckman J. F. Dolnansky E. S. Hardin M. T. Clubb M. Walkup J. T. Stevenson J.. (1990). Perinatal factors in the expression of Tourette's syndrome: an exploratory study. J. Am. Acad. Child Adolesc. Psychiatry 29, 220–226. 10.1097/00004583-199003000-00010 1969861

[B95] Leckman J. F. Katsovich L. Kawikova I. Lin H. Zhang H. Krönig H.. (2005). Increased serum levels of interleukin-12 and tumor necrosis factor-alpha in Tourette's syndrome. Biol. Psychiatry 57, 667–673. 10.1016/j.biopsych.2004.12.004 15780855

[B96] Leckman J. F. Price R. A. Walkup J. T. Ort S. Pauls D. L. Cohen D. J. (1987). Nongenetic factors in Gilles de la Tourette's syndrome. Arch. Gen. Psychiatry 44:100. 3467660 10.1001/archpsyc.1987.01800130112025

[B97] Lécuyer M. A. Kebir H. Prat A. (2016). Glial influences on BBB functions and molecular players in immune cell trafficking. Biochim. Biophys. Acta 1862, 472–482. 10.1016/j.bbadis.2015.10.004 26454208

[B98] Levine S. Saltzman A. (1987). Nonspecific stress prevents relapses of experimental allergic encephalomyelitis in rats. Brain Behav. Immun. 1, 336–341. 3502584 10.1016/0889-1591(87)90036-5

[B99] Levine S. Strebel R. Wenk E. J. Harman P. J. (1962a). Suppression of experimental allergic encephalomyelitis by stress. Proc. Soc. Exp. Biol. Med. 109, 294–298. 14464656 10.3181/00379727-109-27183

[B100] Levine S. Wenk E. J. Muldoon T. N. Cohen S. G. (1962b). Enhancement of experimental allergic encephalomyelitis by adrenalectomy. Proc. Soc. Exp. Biol. Med. 111, 383–385. 13930175 10.3181/00379727-111-27799

[B101] Levite M. (2014). Glutamate receptor antibodies in neurological diseases: anti-AMPA-GluR3 antibodies, anti-NMDA-NR1 antibodies, anti-NMDA-NR2A/B antibodies, anti-mGluR1 antibodies or anti-mGluR5 antibodies are present in subpopulations of patients with either: epilepsy, encephalitis, cerebellar ataxia, systemic lupus erythematosus (SLE) and neuropsychiatric SLE, Sjogren's syndrome, schizophrenia, mania or stroke. These autoimmune anti-glutamate receptor antibodies can bind neurons in few brain regions, activate glutamate receptors, decrease glutamate receptor's expression, impair glutamate-induced signaling and function, activate blood brain barrier endothelial cells, kill neurons, damage the brain, induce behavioral/psychiatric/cognitive abnormalities and ataxia in animal models, and can be removed or silenced in some patients by immunotherapy. J. Neural Transm. 121, 1029–1075. 10.1007/s00702-014-1193-3 25081016

[B102] Levite M. Hermelin A. (1999). Autoimmunity to the glutamate receptor in mice–a model for Rasmussen's encephalitis? J. Autoimmun. 13, 73–82. 10.1006/jaut.1999.0297 10441170

[B103] Levite M. Fleidervish I. A. Schwarz A. Pelled D. Futerman A. H. (1999). Autoantibodies to the glutamate receptor kill neurons via activation of the receptor ion channel. J. Autoimmun. 13, 61–72. 10.1006/jaut.1999.0301 10441169

[B104] Loiselle C. R. Lee O. Moran T. H. Singer H. S. (2004). Striatal microinfusion of Tourette syndrome and PANDAS sera: failure to induce behavioral changes. Mov. Disord. 19, 390–396. 10.1002/mds.10522 15077236

[B105] Lombroso P. J. Scahill L. (2008). Tourette syndrome and obsessive-compulsive disorder. Brain Dev. 30, 231–237. 10.1016/j.braindev.2007.09.001 17937978 PMC2291145

[B106] Lotan D. Benhar I. Alvarez K. Mascaro-Blanco A. Brimberg L. Frenkel D.. (2014). Behavioral and neural effects of intra-striatal infusion of anti-streptococcal antibodies in rats. Brain Behav. Immun. 38, 249–262. 10.1016/j.bbi.2014.02.009 24561489 PMC4000697

[B107] Lyons D. M. Macrì S. (2011). Resilience and adaptive aspects of stress in neurobehavioral development. Neurosci. Biobehav. Rev. 35:1451. 10.1016/j.neubiorev.2010.09.004 20851144

[B108] Ma Y. Mehta S. L. Lu B. Li P. A. (2011). Deficiency in the inner mitochondrial membrane peptidase 2-like (Immp21) gene increases ischemic brain damage and impairs mitochondrial function. Neurobiol. Dis. 44, 270–276. 10.1016/j.nbd.2011.06.019 21824519 PMC3185146

[B109] Macrì S. Ceci C. Onori M. P. Invernizzi R. W. Bartolini E. Altabella L.. (2015). Mice repeatedly exposed to Group-A beta-Haemolytic Streptococcus show perseverative behaviors, impaired sensorimotor gating, and immune activation in rostral diencephalon. Sci. Rep. 5:13257. 10.1038/srep13257 26304458 PMC4548234

[B110] Macrì S. Proietti Onori M. Laviola G. (2013). Theoretical and practical considerations behind the use of laboratory animals for the study of Tourette syndrome. Neurosci. Biobehav. Rev. 37, 1085–1100. 10.1016/j.neubiorev.2013.03.014 23583771

[B111] Mandolesi G. Grasselli G. Musumeci G. Centonze D. (2010). Cognitive deficits in experimental autoimmune encephalomyelitis: neuroinflammation and synaptic degeneration. Neurol. Sci. 31(Suppl. 2), S255–S259. 10.1007/s10072-010-0369-3 20635112

[B112] Mansbach R. S. Geyer M. A. Braff D. L. (1988). Dopaminergic stimulation disrupts sensorimotor gating in the rat. Psychopharmacology 94, 507–514. 3131796 10.1007/BF00212846

[B113] Margari F. Petruzzelli M. G. Mianulli R. Toto M. Pastore A. Bizzaro N.. (2013). Anti-brain autoantibodies in the serum of schizophrenic patients: a case-control study. Psychiatry Res. 210, 800–805. 10.1016/j.psychres.2013.09.006 24103908

[B114] Marques-Dias M. J. Mercadante M. T. Tucker D. Lombroso P. (1997). Sydenham's chorea. Psychiatr. Clin. North Am. 20, 809–820. 9443351 10.1016/s0193-953x(05)70346-4

[B115] Martino D. Chiarotti F. Buttiglione M. Cardona F. Creti R. Nardocci N.. (2011). The relationship between group A streptococcal infections and Tourette syndrome: a study on a large service-based cohort. Dev. Med. Child Neurol. 53, 951–957. 10.1111/j.1469-8749.2011.04018.x 21679362

[B116] Martino D. Dale R. C. Gilbert D. L. Giovannoni G. Leckman J. F. (2009). Immunopathogenic mechanisms in tourette syndrome: a critical review. Mov. Disord. 24, 1267–1279. 10.1002/mds.22504 19353683 PMC3972005

[B117] Martino D. Zis P. Buttiglione M. (2015). The role of immune mechanisms in Tourette syndrome. Brain Res. 1617, 126–143. 10.1016/j.brainres.2014.04.027 24845720

[B118] Meagher M. W. Sieve A. N. Johnson R. R. Satterlee D. Belyavskyi M. Mi W.. (2010). Neonatal maternal separation alters immune, endocrine, and behavioral responses to acute Theiler's virus infection in adult mice. Behav. Genet. 40, 233–249. 10.1007/s10519-010-9333-5 20135342 PMC2862313

[B119] Meldrum B. S. (2000). Glutamate as a neurotransmitter in the brain: review of physiology and pathology. J. Nutr. 130(4S Suppl), 1007S–1015S. 10736372 10.1093/jn/130.4.1007S

[B120] Morris C. M. Pardo-Villamizar C. Gause C. D. Singer H. S. (2009). Serum autoantibodies measured by immunofluorescence confirm a failure to differentiate PANDAS and Tourette syndrome from controls. J. Neurol. Sci. 276, 45–48. 10.1016/j.jns.2008.08.032 18823914

[B121] Murphy T. K. Kurlan R. Leckman J. (2010). The immunobiology of Tourette's disorder, pediatric autoimmune neuropsychiatric disorders associated with Streptococcus, and related disorders: a way forward. J. Child Adolesc. Psychopharmacol. 20, 317–331. 10.1089/cap.2010.0043 20807070 PMC4003464

[B122] Nordstrom E. J. Burton F. H. (2002). A transgenic model of comorbid Tourette's syndrome and obsessive-compulsive disorder circuitry. Mol. Psychiatry 7, 617–625, 524. 10.1038/sj.mp.4001144 12140785

[B123] Olechowski C. J. Tenorio G. Sauve Y. Kerr B. J. (2013). Changes in nociceptive sensitivity and object recognition in experimental autoimmune encephalomyelitis (EAE). Exp. Neurol. 241, 113–121. 10.1016/j.expneurol.2012.12.012 23291347

[B124] Park H. S. Francis K. P. Yu J. Cleary P. P. (2003). Membranous cells in nasal-associated lymphoid tissue: a portal of entry for the respiratory mucosal pathogen group A streptococcus. J. Immunol. 171, 2532–2537. 10.4049/jimmunol.171.5.2532 12928403

[B125] Petek E. Windpassinger C. Vincent J. B. Cheung J. Boright A. P. Scherer S. W.. (2001). Disruption of a novel gene (IMMP2L) by a breakpoint in 7q31 associated with Tourette syndrome. Am. J. Hum. Genet. 68, 848–858. 10.1086/319523 11254443 PMC1275638

[B126] Platt S. R. (2007). The role of glutamate in central nervous system health and disease–a review. Vet. J. 173, 278–286. 10.1016/j.tvjl.2005.11.007 16376594

[B127] Podolska M. J. Biermann M. H. Maueröder C. Hahn J. Herrmann M. (2015). Inflammatory etiopathogenesis of systemic lupus erythematosus: an update. J. Inflamm. Res. 8, 161–171. 10.2147/JIR.S70325 26316795 PMC4548750

[B128] Poliak S. Gollan L. Martinez R. Custer A. Einheber S. Salzer J. L.. (1999). Caspr2, a new member of the neurexin superfamily, is localized at the juxtaparanodes of myelinated axons and associates with K+ channels. Neuron 24, 1037–1047. 10624965 10.1016/s0896-6273(00)81049-1

[B129] Proenca C. C. Gao K. P. Shmelkov S. V. Rafii S. Lee F. S. (2011). Slitrks as emerging candidate genes involved in neuropsychiatric disorders. Trends Neurosci. 34, 143–153. 10.1016/j.tins.2011.01.001 21315458 PMC3051006

[B130] Rabchevsky A. G. Degos J. D. Dreyfus P. A. (1999). Peripheral injections of Freund's adjuvant in mice provoke leakage of serum proteins through the blood-brain barrier without inducing reactive gliosis. Brain Res. 832, 84–96. 10375654 10.1016/s0006-8993(99)01479-1

[B131] Rickard M. D. (2004). The use of animals for research on animal diseases: its impact on the harm-benefit analysis. Altern. Lab. Anim. 32(Suppl. 1A), 225–227. 23577464 10.1177/026119290403201s37

[B132] Rizzo R. Gulisano M. Pavone P. Fogliani F. Robertson M. M. (2006). Increased antistreptococcal antibody titers and anti-basal ganglia antibodies in patients with Tourette syndrome: controlled cross-sectional study. J. Child Neurol. 21, 747–753. 10.1177/08830738060210091001 16970879

[B133] Roque S. Mesquita A. R. Palha J. A. Sousa N. Correia-Neves M. (2014). The behavioral and immunological impact of maternal separation: a matter of timing. Front. Behav. Neurosci. 8:192. 10.3389/fnbeh.2014.00192 24904343 PMC4033212

[B134] Russig H. Spooren W. Durkin S. Feldon J. Yee B. K. (2004). Apomorphine-induced disruption of prepulse inhibition that can be normalised by systemic haloperidol is insensitive to clozapine pretreatment. Psychopharmacology 175, 143–147. 10.1007/s00213-004-1810-1 14985922

[B135] Scahill L. Erenberg G. Berlin C. M. Jr. Budman C. Coffey B. J. Jankovic J.. (2006). Contemporary assessment and pharmacotherapy of Tourette syndrome. NeuroRx 3, 192–206. 10.1016/j.nurx.2006.01.009 16554257 PMC3593444

[B136] Schlumberger W. Hornig N. Lange S. Probst C. Komorowski L. Fechner K.. (2014). Differential diagnosis of membranous nephropathy with autoantibodies to phospholipase A2 receptor 1. Autoimmun. Rev. 13, 108–113. 10.1016/j.autrev.2013.09.005 24075959

[B137] Scoriels L. Salek R. M. Goodby E. Grainger D. Dean A. M. West J. A.. (2015). Behavioural and molecular endophenotypes in psychotic disorders reveal heritable abnormalities in glutamatergic neurotransmission. Transl. Psychiatry 5:e540. 10.1038/tp.2015.26 25826115 PMC4429170

[B138] Sengupta A. Winters B. Bagley E. E. McNally G. P. (2016). Disrupted prediction error links excessive amygdala activation to excessive fear. J. Neurosci. 36, 385–395. 10.1523/JNEUROSCI.3670-15.2016 26758831 PMC6602025

[B139] Shemer A. Jung S. (2015). Differential roles of resident microglia and infiltrating monocytes in murine CNS autoimmunity. Semin. Immunopathol. 37, 613–623. 10.1007/s00281-015-0519-z 26240063

[B140] Shepshelovich D. Shoenfeld Y. (2006). Prediction and prevention of autoimmune diseases: additional aspects of the mosaic of autoimmunity. Lupus 15, 183–190. 10.1191/0961203306lu2274rr 16634374

[B141] Shprecher D. Kurlan R. (2009). The management of tics. Mov. Disord. 24, 15–24. 10.1002/mds.22378 19170198 PMC2701289

[B142] Silberberg D. Anand N. P. Michels K. Kalaria R. N. (2015). Brain and other nervous system disorders across the lifespan - global challenges and opportunities. Nature 527, S151–S154. 10.1038/nature16028 26580320

[B143] Singer H. S. Hong J. J. Yoon D. Y. Williams P. N. (2005a). Serum autoantibodies do not differentiate PANDAS and Tourette syndrome from controls. Neurology 65, 1701–1707. 10.1212/01.wnl.0000183223.69946.f1 16207842

[B144] Singer H. S. Mink J. W. Loiselle C. R. Burke K. A. Ruchkina I. Morshed S.. (2005b). Microinfusion of antineuronal antibodies into rodent striatum: failure to differentiate between elevated and low titers. J. Neuroimmunol. 163, 8–14. 10.1016/j.jneuroim.2005.02.018 15885303

[B145] Stanwood G. D. Lucki I. McGonigle P. (2000). Differential regulation of dopamine D2 and D3 receptors by chronic drug treatments. J. Pharmacol. Exp. Ther. 295, 1232–1240. 11082460

[B146] State M. W. (2011). The genetics of Tourette disorder. Curr. Opin. Genet. Dev. 21, 302–309. 10.1016/j.gde.2011.01.007 21277193 PMC3102152

[B147] Stillman A. A. Krsnik Z. Sun J. Rasin M. R. State M. W. Sestan N.. (2009). Developmentally regulated and evolutionarily conserved expression of SLITRK1 in brain circuits implicated in Tourette syndrome. J. Comp. Neurol. 513, 21–37. 10.1002/cne.21919 19105198 PMC3292218

[B148] Swanborg R. H. (2001). Experimental autoimmune encephalomyelitis in the rat: lessons in T-cell immunology and autoreactivity. Immunol. Rev. 184, 129–135. 10.1034/j.1600-065x.2001.1840112.x 12086308

[B149] Swedo S. E. Grant P. J. (2005). Annotation: PANDAS: a model for human autoimmune disease. J. Child Psychol. Psychiatry 46, 227–234. 10.1111/j.1469-7610.2004.00386.x 15755299

[B150] Swedo S. E. Leonard H. L. Garvey M. Mittleman B. Allen A. J. Perlmutter S.. (1998). Pediatric autoimmune neuropsychiatric disorders associated with streptococcal infections: clinical description of the first 50 cases. Am. J. Psychiatry 155, 264–271. 9464208 10.1176/ajp.155.2.264

[B151] Swedo S. E. Leonard H. L. Schapiro M. B. Casey B. J. Mannheim G. B. Lenane M. C.. (1993). Sydenham's chorea: physical and psychological symptoms of St Vitus dance. Pediatrics 91, 706–713. 8464654

[B152] Swedo S. E. Rapoport J. L. Cheslow D. L. Leonard H. L. Ayoub E. M. Hosier D. M.. (1989). High prevalence of obsessive-compulsive symptoms in patients with Sydenham's chorea. Am. J. Psychiatry 146, 246–249. 10.1176/ajp.146.2.246 2912267

[B153] Swerdlow N. R. (2013). Update: studies of prepulse inhibition of startle, with particular relevance to the pathophysiology or treatment of Tourette Syndrome. Neurosci. Biobehav. Rev. 37, 1150–1156. 10.1016/j.neubiorev.2012.09.002 23017868 PMC3566359

[B154] Swerdlow N. R. Sutherland A. N. (2005). Using animal models to develop therapeutics for Tourette Syndrome. Pharmacol. Ther. 108, 281–293. 10.1016/j.pharmthera.2005.05.003 15970330

[B155] Swerdlow N. R. Benbow C. H. Zisook S. Geyer M. A. Braff D. L. (1993). A preliminary assessment of sensorimotor gating in patients with obsessive compulsive disorder. Biol. Psychiatry 33, 298–301. 8471686 10.1016/0006-3223(93)90300-3

[B156] Swerdlow N. R. Geyer M. A. Braff D. L. (2001a). Neural circuit regulation of prepulse inhibition of startle in the rat: current knowledge and future challenges. Psychopharmacology 156, 194–215. 10.1007/s002130100799 11549223

[B157] Swerdlow N. R. Karban B. Ploum Y. Sharp R. Geyer M. A. Eastvold A. (2001b). Tactile prepuff inhibition of startle in children with Tourette's syndrome: in search of an “fMRI-friendly” startle paradigm. Biol. Psychiatry 50, 578–585. 10.1016/S0006-3223(01)01164-7 11690592

[B158] Swerdlow N. R. Light G. A. Cadenhead K. S. Sprock J. Hsieh M. H. Braff D. L. (2006). Startle gating deficits in a large cohort of patients with schizophrenia: relationship to medications, symptoms, neurocognition, and level of function. Arch. Gen. Psychiatry 63, 1325–1335. 10.1001/archpsyc.63.12.1325 17146007

[B159] Swerdlow N. R. Paulsen J. Braff D. L. Butters N. Geyer M. A. Swenson M. R. (1995). Impaired prepulse inhibition of acoustic and tactile startle response in patients with Huntington's disease. J. Neurol. Neurosurg. Psychiatr. 58, 192–200. 7876851 10.1136/jnnp.58.2.192 PMC1073317

[B160] Taylor J. R. Morshed S. A. Parveen S. Mercadante M. T. Scahill L. Peterson B. S.. (2002). An animal model of Tourette's syndrome. Am. J. Psychiatry 159, 657–660. 10.1176/appi.ajp.159.4.657 11925307

[B161] Valls-Sole J. Munoz J. E. Valldeoriola F. (2004). Abnormalities of prepulse inhibition do not depend on blink reflex excitability: a study in Parkinson's disease and Huntington's disease. Clin. Neurophysiol. 115, 1527–1536. 10.1016/j.clinph.2004.02.014 15203054

[B162] van der Staay F. J. (2006). Animal models of behavioral dysfunctions: basic concepts and classifications, and an evaluation strategy. Brain Res. Rev. 52, 131–159. 10.1016/j.brainresrev.2006.01.006 16529820

[B163] van der Staay F. J. Arndt S. S. Nordquist R. E. (2009). Evaluation of animal models of neurobehavioral disorders. Behav. Brain Funct. 5:11. 10.1186/1744-9081-5-11 19243583 PMC2669803

[B164] Verkerk A. J. Mathews C. A. Joosse M. Eussen B. H. Heutink P. Oostra B. A.. (2003). CNTNAP2 is disrupted in a family with Gilles de la Tourette syndrome and obsessive compulsive disorder. Genomics 82, 1–9. 10.1016/S0888-7543(03)00097-1 12809671

[B165] Verschuuren J. J. Huijbers M. G. Plomp J. J. Niks E. H. Molenaar P. C. Martinez-Martinez P.. (2013). Pathophysiology of myasthenia gravis with antibodies to the acetylcholine receptor, muscle-specific kinase and low-density lipoprotein receptor-related protein 4. Autoimmun. Rev. 12, 918–923. 10.1016/j.autrev.2013.03.001 23535160

[B166] Wick G. Hu Y. Schwarz S. Kroemer G. (1993). Immunoendocrine communication via the hypothalamo-pituitary-adrenal axis in autoimmune diseases. Endocr. Rev. 14, 539–563. 10.1210/edrv-14-5-539 8262005

[B167] Willner P. (1984). The validity of animal models of depression. Psychopharmacology 83, 1–16. 6429692 10.1007/BF00427414

[B168] Willner P. Mitchell P. J. (2002). The validity of animal models of predisposition to depression. Behav. Pharmacol. 13, 169–188. 10.1097/00008877-200205000-00001 12122308

[B169] Yaddanapudi K. Hornig M. Serge R. De Miranda J. Baghban A. Villar G.. (2010). Passive transfer of streptococcus-induced antibodies reproduces behavioral disturbances in a mouse model of pediatric autoimmune neuropsychiatric disorders associated with streptococcal infection. Mol. Psychiatry 15, 712–726. 10.1038/mp.2009.77 19668249

[B170] Yeh C. B. Shui H. A. Chu T. H. Chen Y. A. Tsung H. C. Shyu J. F. (2012). Hyperpolarisation-activated cyclic nucleotide channel 4 (HCN4) involvement in Tourette's syndrome autoimmunity. J. Neuroimmunol. 250, 18–26. 10.1016/j.jneuroim.2012.05.009 22683190

[B171] Zebardast N. Crowley M. J. Bloch M. H. Mayes L. C. Wyk B. V. Leckman J. F.. (2013). Brain mechanisms for prepulse inhibition in adults with Tourette syndrome: initial findings. Psychiatry Res. 214, 33–41. 10.1016/j.pscychresns.2013.05.009 23916249 PMC3932431

